# Restrictions on indoor and outdoor NO_2_ emissions to reduce disease burden for pediatric asthma in China: A modeling study

**DOI:** 10.1016/j.lanwpc.2022.100463

**Published:** 2022-05-04

**Authors:** Ying Hu, John S. Ji, Bin Zhao

**Affiliations:** a Department of Building Science, School of Architecture, Tsinghua University, Beijing 100084, China; b Vanke School of Public Health, Tsinghua University, Beijing 100084, China; c Beijing Key Laboratory of Indoor Air Quality Evaluation and Control, Tsinghua University, Beijing 100084, China

**Keywords:** Nitrogen dioxide, Pediatric asthma, Cooking, Ambient, China

## Abstract

**Background:**

Epidemiological studies have reported the associations between nitrogen dioxide (NO_2_) and pediatric asthma incidence, but unable to ascertain indoor NO_2_ sources. We estimated the pediatric asthma incidence and corresponding economic losses attributable to NO_2_ from indoor and outdoor sources in urban areas in China.

**Methods:**

Exposure to NO_2_ from indoor and outdoor sources in 2019 were estimated separately with a source-specific model validated by measurements from different studies, and NO_2_ exposure after restricting emissions indoor (from cooking or second-hand smoking) and outdoor (to meet WHO interim targets and air quality guideline) were projected. Disease burden of NO_2_-attributable new-onset pediatric asthma were estimated based on NO_2_ exposure, concentration-response function from a meta-analysis, and number of pediatric asthma populations. Economic impacts were estimated based on the costs of pediatric asthma in China.

**Findings:**

In China, NO_2_ is associated with an estimated 637,000 (95% uncertainty interval 358,000–851,000) new pediatric asthma cases and 1,358 million (674–2145) RMB economic losses in urban areas in 2019. 296,000 (222,000–523,000) new pediatric asthma cases would be prevented each year by restricting NO_2_ emissions indoor, i.e., switching from using gas stoves to electic stoves for cooking. 393,000 (119,000–463,000) new pediatric asthma cases would be prevented each year when outdoor air meets the air quality guideline for NO_2_ (< 10 µg/m^3^).

**Interpretation:**

Restricting both indoor and outdoor NO_2_ emissions are necessary to reduce pediatric asthma incidence in urban areas. NO_2_ restrictions may be achieved through clean energy transition and adoption of climate change mitigation activities.

**Funding:**

Vanke School of Public Health, Tsinghua University (2021JC005).

**Research in context**

**Evidence before this study**

We searched Web of Science and PubMed with no language restriction, from January 1, 1999 to January 4, 2022, using the search terms (“nitrogen dioxide” AND (“disease burden” OR “indoor”)). Epidemiological studies have reported a significant association between ambient nitrogen dioxide (NO_2_), recognized as a proxy of traffic-related air pollution, and pediatric asthma, the most common chronic disease among children worldwide. There are also evident sources of NO_2_ indoor even in areas with no use of solid fuels and kerosene, i.e., NO_2_ produced during the combustion of tobacco and gaseous fuels. Latest study has demonstrated the comparable contributions of NO_2_ from indoor and outdoor sources in the air people breath. However, no studies focused on the disease burden of NO_2_ from indoor sources, let alone the health benefits of restrictions on indoor NO_2_ emissions.

**Added value of this study**

The uniqueness of this study is estimating the disease burden attributable to exposure to NO_2_ from indoor and outdoor sources separately; and comparing the health benefits of restricting indoor and outdoor NO_2_ emissions. We focused on urban areas in China, where facing serious air pollution of NO_2_ both indoor and outdoor. NO_2_ is estimated to attribute to hundreds of thousands of pediatric asthma cases in Chinese urban areas in 2019, with significantly contributions of NO_2_ from both indoor and outdoor sources. The cost associated with asthma of each child with asthma is equivalent to about 5% per capita disposable income in China. The reduction of pediatric asthma cases by restricting indoor NO_2_ emissions, i.e., switching from using gas stoves to electric stoves for cooking, is comparable to the burden reduction when outdoor air meets WHO air quality guideline issued in 2021.

**Implications of the available evidence**

This study demonstrates the importance of restricting indoor and outdoor NO_2_ emissions to reduce the disease burden attributable to NO_2_, typically in pediatric asthma incidence. The measurements to restrict indoor and outdoor NO_2_ emissions, i.e., switching from using gas stove to electric stove for cooking and switching the traditional fossil energy vehicles to new energy vehicles, reflect the demand to adjust the energy consumption structure. These actions may be a win–win opportunity for countries facing both climate change and air pollution challenges.

Alt-text: Unlabelled box

## Introduction

Air pollution is one of the top five risk factors for disease burden around the world according to the latest estimates from the Global Burden of Diseases, Injuries, and Risk Factors Study.^1^ Reducing disease burden attributed to air pollution is also a key indicator of the United Nations Sustainable Development Goal 3, to ensure healthy lives and promote well-being for all at all ages, as well as Goal 11 for sustainable cities and communities.^2^ Long-term exposure to high levels of air pollutions would lead to a variety of non-communicable diseases, particularly for children.^1^ Asthma is the most common chronic disease among children worldwide, with rising incidences in developing economies.^3^ While air pollution control measures have been haphazardly effective in controlling particulate matter exposures, anthropogenic sources of NO_2_ mainly derived from include on-road and non-road transportation tailpipe emissions may not correlate with particulate matter control measures. There are recent advancements in our understanding of NO_2_ and onset of allergic diseases. Epidemiological studies have provided evidences of the associations between air pollutants and new-onset asthma in children. The evidence for other air pollutants (e.g., fine particulate matter) is mixed except for nitrogen dioxide (NO_2_).^4^^,^^5^ Epidemiological studies in Europe, North America, Japan, China, Korea have consistently shown a significant positive association between NO_2_ and pediatric asthma incidence.^4^ Achakulwisut et al.^6^ have linked NO_2_ with about 4·0 million annual number of new pediatric asthma cases in 194 countries. And China has been estimated to have the most considerable national burdens of NO_2_-attributable pediatric asthma, with 760,000 new cases per year and more than 50% of the cases occurring in urban areas.^6^

The World Health Organization (WHO) issued new Air Quality Guidelines (AQG) on Sept 22, 2021 (WHO AQG 2021), setting AQG for NO_2_ of annual averaged concentration of 10 μg/m^3^ and applied for both indoor and outdoor air.^7^ NO_2_ is usually thought of as a proxy of traffic-related air pollution. Whereas there are also evident sources of NO_2_ indoor, i.e., NO_2_ produced during combustion processes indoors, such as cooking (i.e., gas combustion) and smoking (i.e., tobacco combustion),^8^ even in areas without using solid fuels and kerosene. Urban areas are typical areas with heavy traffic and do not use solid fuels and kerosene, especially in urban China where the population density is extremely high. Our latest study figured out indoor sources contribute ∼33% to human exposure to NO_2_ in Chinese urban.^9^ Despite the importance of indoor sources in NO_2_ exposure, to the best of our knowledge, no studies focus on the disease burden of NO_2_ from indoor sources,^6^^,^^8^ let alone the policies to restrict NO_2_ emitted indoors. This is unique compared with those previous studies focused on outdoor NO_2_. The formulation of policies to restrict the NO_2_ produced indoor, such as implementing a smoking ban and using electric stoves for cooking, may well be an essential initiative to reduce the disease burden attributable to NO_2_ exposure.

In this study, we estimated the number of pediatric asthma cases and corresponding economic losses attributable to exposure to NO_2_ from indoor and outdoor sources in Chinese urban areas in 2019. We further projected and compared the health benefits and corresponding economic benefits of policies on restricting the NO_2_ emissions indoor and outdoor.

## Methods

### Overview

**Figure 1:**
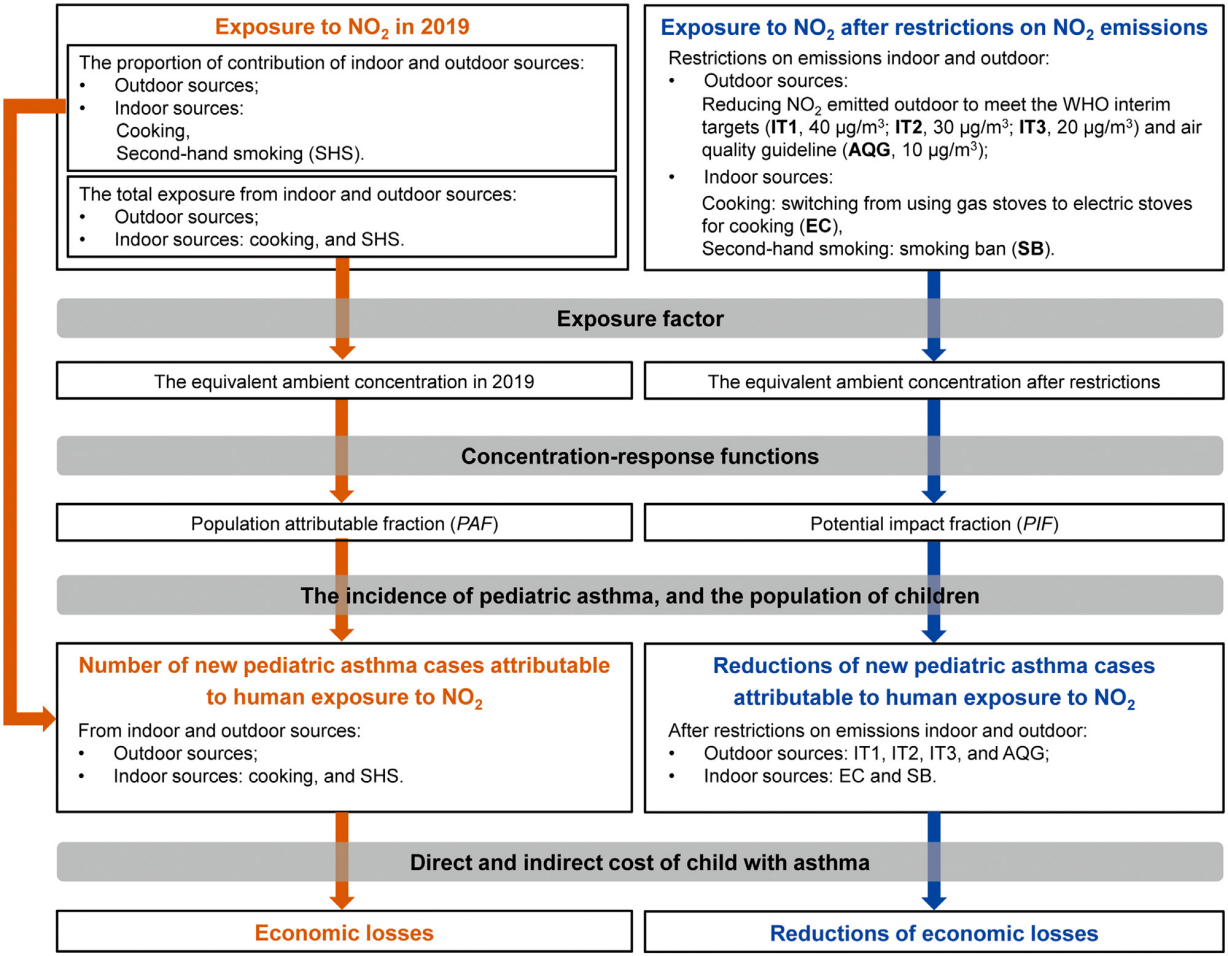
Methodology framework.

We first generated the concentration of children exposure to NO_2_ from indoor and outdoor sources in urban areas in China in 2019 from a previous modeling study (boys and girls in 6 age groups, i.e., 0–0·5 y, 0·5–1 y, 1-2 y, 3–6 y, 7–11 y, 12–17 y, from 330 Chinese cities).^9^ We estimated the NO_2_ exposure after restricting NO_2_ emissions from indoor and outdoor sources. We then explored pediatric asthma incidence attributable to NO_2_ from indoor and outdoor sources for urban areas in China in 2019. We projected the reduction of pediatric asthma incidence after restrictions on NO_2_ emissions. We also estimated the economic burden associated with NO_2_-attributable pediatric asthma incidence with the cost of illness methods. The methodology framework is shown in Figure 1. The uncertainty interval (UI) of the results was obtained by Monte Carlo method.

### Exposure to NO_2_ from different sources in 2019

For human exposure to outdoor sources, it is the sum of exposure to outdoor originated NO_2_ indoor when people stay indoors and exposure to outdoor NO_2_ directly when people stay outdoor. Human exposure to indoor sources is the exposure to NO_2_ emitted from gas combustion during cooking and tobacco combustion during smoking. The concentration of NO_2_ exposure is defined as the average concentration of NO_2_ in the air people breathe during the studied period, reporting in µg/m^3^. We estimated the concentration of human exposure to NO_2_ from indoor and outdoor sources in Chinese urban areas in 2019 based on a validated source-specific model in our previous study.^9^ The model and its validation were detailed in the appendix (**Note S1** and **Figure S1**). The annual average concentration of human exposure to NO_2_ from indoor and outdoor sources for children of 6 age groups (i.e., 0–0·5, 0·5–1, 1-2, 3–6, 7–11, 12–17 years old) and two genders (boys and girls) and exposure to second-hand smoke or not (from smoking and non-smoking households) in urban areas in 330 Chinese cities in 2019 were provided in Table S13 in Hu and Zhao.^9^ Mean and standard deviation in Table S13 in Hu and Zhao^9^ indicates the intra-population variability distribution of NO_2_ exposure.

The annual average concentration of NO_2_ exposure from cooking, second-hand smoking and outdoor sources were represented by the parameters *C_cooking_, C_SHS_* and *C_ambient_*, respectively. *C_cooking_* and *C_ambient_* in 2019 were provided as IEC (indoor source exposure concentration) and OEC (outdoor source exposure concentration) for non-smoking households in Table S13 in Hu and Zhao.^9^ *C_SHS_* for people from smoking households in 2019 was calculated with the following equation:

(1) $$C_{SHS}=C_{indoor,\;smoking\;household}-C_{cooking}$$

where *C_indoor,smoking household_* is the annual average concentration of people from smoking households exposed to NO_2_ from indoor sources. *C_indoor,smoking household_* in Chinese urban areas in 2019 was provided as IEC for smoking households in Table S13 in Hu and Zhao.^9^ The annual average concentration of human exposure to NO_2_ from all sources were represented by *C_exp_. C_exp_* for people from non-smoking households were the sum of *C_cooking_* and *C_ambient_* and it is the sum of *C_cooking_, C_SHS_* and *C_ambient_* for people from smoking households.

The proportion of the contribution of cooking, second-hand smoke, and outdoor sources in NO_2_ exposure for a population of specific age groups (6 age groups, i.e., 0–0·5, 0·5–1, 1-2, 3–6, 7–11 and 12–17) and sex groups (boys and girls) in specific cities (330 Chinese cities) were calculated with the following equations:

(2) $$PS_{ambient}=\frac{\bar{C_{ambient}}}{\bar{C_{cooking}}+\bar{C_{ambient}}+P_{SHS}\times \bar{C_{SHS}}}$$

(3) $$PS_{cooking}=\frac{\bar{C_{cooking}}}{\bar{C_{cooking}}+\bar{C_{ambient}}+P_{SHS}\times \bar{C_{SHS}}}$$

(4) $$PS_{SHS}=\frac{P_{SHS}\times \bar{C_{SHS}}}{\bar{C_{cooking}}+\bar{C_{ambient}}+P_{SHS}\times \bar{C_{SHS}}}$$

*PS* is the proportion of the contribution of different sources in NO_2_ exposure, the subscripts *ambient, cooking* and *SHS* of *PS* are the outdoor sources, cooking, and second-hand smoke. $\bar{C_{ambient}}$, $\bar{C_{cooking}}$, and $\bar{C_{SHS}}$ are the mean of *C_ambient_, C_cooking_*, and *C_SHS_*, respectively. *P_SHS_* is the proportion of the population exposed to second-hand smoke. The details of calculation of *P_SHS_* were provided in the appendix (**Note S2**). The value of *PS* in 2019 were shown in appendix (**Figure S2**).

### NO_2_ exposure after restrictions

The restrictions on indoor and outdoor NO_2_ emissions included a smoking ban, switching from using a gas stove to electric stove for cooking, and reducing NO_2_ emitted outdoor. To see the scope for health benefits from these policies, we projected the scenarios where these policies were 100% achieved (**Table S1** in appendix):

smoking ban (SB): no people smoking, to reduce the NO_2_ produced by tobacco combustion during smoking (*P_SHS_*=0; *C_cooking_* and *C_ambient_* were equal to that in 2019);
switching from using a gas stove to electric stove for cooking (EC): all residents using electric stove for cooking in Chinese urban areas, to reduce the NO_2_ produced by gas combustion during cooking (*C_cooking_*=0; *C_smoking_* and *C_ambient_* were equal to that in 2019);
reducing NO_2_ emitted outdoor: the outdoor air meets the WHO interim targets (IT) and AQG for annual NO_2_ concentration issued in 2021^7^ (*C_cooking_* and *C_smoking_* were equal to that in 2019). The *C_ambient_* when IT1, IT2, IT3 and AQG were calculated by
  (5) $$\begin{array}{c} C_{ambient}=\left\{ \begin{array}{c} C_{ambient}\;in\;2019\;\;\;\;\;\;\;\;\;\;\;\;\;for\;Outdoor\;concentration\;in\;2019\leq Target\\ Target\times f_{exp}\;\;\;\;\;\;\;\;\;\;\;\;\;\;\;\;\;\;for\;Outdoor\;concentration\;in\;2019>Target \end{array}\right. \end{array}$$

in which *Target* is the target concentration of NO_2_ outdoor, and it is 40 μg/m^3^ (IT1), 30 μg/m^3^ (IT2), 20 μg/m^3^ (IT3), or 10 μg/m^3^ (AQG) in this study. *f_exp_* is the exposure factor. When outdoor NO_2_ enters the indoor environment, some of the NO_2_ will be removed by indoor surfaces due to deposition or chemical reaction, resulting in a lower concentration of outdoor-originated NO_2_ in indoor environment compared with the concentration of NO_2_ outdoor. Besides, there exist variations of outdoor activities, respiratory rate, building ventilation and indoor activities between different populations. *f_exp_* is a parameter to modify human exposure to ambient NO_2_ considering building ventilation, air exchange rate between indoor and outdoor environments, indoor surface removal due to deposition or chemical reaction, duration of outdoor activities and respiratory rate for different populations.^10^ The *f_exp_* of NO_2_ in 31 provinces in China was used in this study and they were estimated and verified in our previous study (**Table S3** in appendix).^10^ In Eq. (5), we assumed the concentration of ambient NO_2_ will remain the same as that in 2019 for areas where outdoor concentration in 2019 could meet the target. The study by Shi et al.^11^ indicate the rural-to-urban migration may increase the emissions of NO_2_ outdoor in urban areas in China, resulting in extra environmental pressure. Considering the combining effect of other anthropogenic emission reductions, we think those areas may be some insensitive and thus our assumption is reasonable.

### Concentration-response relationships

The risks of pediatric asthma associated with long‐term exposure to NO_2_ have been described using concentration‐response relationships. Khreis et al.^4^ reviewed 41 studies published between 1999 and 2016 (20 studies from Europe, 11 from North America, five from Japan, three from China, one from South Korea, and one from Taiwan) to identify meta-analyses of epidemiological studies on ambient NO_2_ and pediatric asthma. Following their approach, relative risks (*RR*) of pediatric asthma incidence attributable to ambient NO_2_ were determined using the following equation:

(6) $$RR=\left\{ \begin{array}{cc} 1 & for\;C\leq LC\\ RR_{0}^{\frac{C-LC}{\Delta C_{0}}} & for\;C>LC \end{array}\right.$$

where *RR* is the relative risk, representing the ratio of the probability of developing asthma for children exposed to NO_2_ to the probability of developing pediatric asthma for children in a comparison group. A comparison group was the group exposed to NO_2_ at the low-concentration threshold (*LC*), below which there is no risk of pediatric asthma development. *RR_0_* represents the change in relative risk for per unit concentration increase (*△C_0_*). *C* is the concentration of ambient NO_2_. Khreis and colleagues^4^ reported an *RR* of 1·26 per 10 ppb ambient NO_2_ (95% uncertainty interval [UI] 1·10–1·37) and assumed LC at 2 ppb (0–5) (1ppb NO_2_ = 0·487 μg/m^3^ NO_2_).

Theoretically, *RR* should be related to the NO_2_ exposure, i.e., the concentration of exposure to NO_2_ from both indoor and outdoor sources, as opposed to ambient concentration. So we multiply the reciprocal of the exposure factor (*1/f_exp_*) by the annual average concentration of human exposure to NO_2_ (*C_exp_*) to obtain the equivalent ambient concentration. Therefore, we related *RR* to the concentrations of NO_2_ exposure using the following equation:

(7) $$RR=\left\{ \begin{array}{cc} 1 & for\;C\leq LC\\ R{R_{0}}^{\frac{C_{exp}/f_{exp}-LC}{\Delta C_{0}}} & for\;C>LC \end{array}\right.$$

### NO_2_-attributable pediatric asthma incidence

We estimated the number of new-onset pediatric asthma cases attributable to long-term exposure to NO_2_ from outdoor source, cooking and second-hand smoke in Chinese urban areas in 2019, and we further projected the reduction of the number of new pediatric asthma cases in the scenarios after restricting NO_2_ emissions.

The number of new pediatric asthma cases attributable to human exposure to NO_2_ from specific sources (*AN_s_*) was determined by the proportion of contribution of specific sources in NO_2_ exposure (*PS_s_*), the population attributable fraction of NO_2_ exposure and incidences of pediatric asthma (*PAF*), the incidence of pediatric asthma (*IR*), and the population of children (*N*). *PAF* refers to the proportion of incidence in a population that can be attributed to a certain risk factor,^1^ i.e., NO_2_ exposure in this study. The new pediatric asthma cases attributable to NO_2_ exposure from specific sources in Chinese urban areas were calculated with the following equation as that applied in the Global Burden of Diseases, Injuries, and Risk Factors Study in 2019^1^:

(8) $$AN_{s}=\sum_{g}\left(PS_{s}\times PAF_{g}\times IR_{g}\times N_{g}\right)$$

where the subscript *s* represents the source of PM_2.5_ and it is *ambient, cooking*, or *SHS* in this study; the subscript *g* represents the population with specific age and sex in specific city. The group-specific population size of children (*N*) in Chinese urban areas in 2019 was calculated based on the urban population of each city in 2019 (**Table S2** in appendix)^12^ and the age and gender composition of children of the population of each province (**Table S5** in appendix).^13^ The age-, sex- and province-specific incidence of pediatric asthma (*IR*) for residences in Chinese urban areas in 2019 (**Table S4** in appendix) was calculated using the age- and sex-specific incidence of pediatric asthma in China in 2019^14^ and the ratio of provincial and regional incidence of pediatric asthma.^15^ *PAF* for the population *g* was calculated by^1^

(9) $$PAF_{g}=\frac{\overset{¯}{RR_{g}}-1}{\bar{RR_{g}}}$$

(10) $$\bar{RR_{g}}=\left(1-P_{SHS,g}\right)\times \bar{RR_{g,non-smoking\;household}}+P_{SHS,g}\times \bar{RR_{g,smoking\;household}}$$

where $\bar{RR_{g}}$ is the average relative risk for the population *g* in 2019, and $\bar{RR_{g,smoking\;household}}$ and $\bar{RR_{g,non-smoking\;household}}$ are the average *RR* for people from smoking and non-smoking households in the population *g*. $\bar{RR_{g,smoking\;household}}$ in 2019 was calculated based on the annual average concentration of exposure to NO_2_ (*C_exp_*) for people from smoking households in 2019 and concentration-response relationships of long‐term NO_2_ exposure and incident of pediatric asthma (Eq. (7)). $\bar{RR_{g,non-smoking\;household}}$ in 2019 was calculated based on *C_exp_* for people from non-smoking households in 2019 and Eq. (7). The value of *RR* in 2019 were shown in appendix (**Table S9**).

The reductions of new pediatric asthma cases attributable to human exposure to NO_2_ from specific sources (*RAN_po_*) were calculated with the following equations^1^:

(11) $$RAN_{po}=\sum_{g}\left(PIF_{po,g}\times IR_{g}\times N_{g}\right)$$

(12) $$PIF_{po,g}=\frac{\bar{RR_{g}}-\bar{RR_{po,g}}}{\bar{RR_{g}}}$$

where *PIF* is potential impact fraction, referring to the proportion reduction in the incidence in a population when a certain risk factor change,^1^ i.e., human exposure to NO_2_ in this study. $\bar{RR_{po,g}}$ is the average relative risk for the population *g* under the scenarios *po* after restrictions on NO_2_ emissions and *po* is SB, EC, IT1, IT2, IT3 or AQG in this study. $\bar{RR_{po,g}}$ were calculated based on NO_2_ exposure (*C_exp_*) after restrictions and concentration-response relationships of long‐term exposure to NO_2_ and asthma incident in children (Eq. (7)). The value of *RR_po_* were provided in appendix (**Table S9**).

### Economic losses

In this study, the cost of illness methods was applied to monetize the health effects of NO_2_ from direct and indirect costs.^16^^,^^17^ Direct costs include the direct costs of treatment and hospitalization related to pediatric asthma. Indirect costs include the reduction in the income due to absence from work for childcare. The total cost in 2019 or reduction of total cost after restrictions on NO_2_ emissions (*Cost*) is defined as:

(13) $$Cost=\left(Cost_{d}+GDP_{p}\times T\right)\times \left(ANorRAN\right)$$

where *Cost_d_* is the per capita direct costs, *GDP_p_* is a daily average of per capita Gross Domestic Product, and *T* is the per capita treatment and hospitalization days. The age- and sex-specific per capita directs cost and the per capita treatment and hospitalization days was from the China Medical Insurance Research Association database (**Table S6** in appendix).^18^ The *GDP_p_* in 330 Chinese cities was from the China city statistical year book-2020 (**Table S2** in appendix).^19^

### Uncertainty analysis

We applied a two-stage Monte Carlo simulation^20^ to obtain the mean and 95% uncertainty interval (95% UI) of the new pediatric asthma cases attributable to NO_2_ exposure and corresponding economic loss or benefits. The two stages reflected the intra-population variability distribution (human exposure to NO_2_), and the uncertainty distribution (the concentration-response function, baseline pediatric asthma incidence, direct cost, and the treatment and hospitalization days), respectively. We first performed 2000 iterations at variability stage (i.e., human exposure to NO_2_) and 1000 iterations at uncertainty stages (i.e., the concentration-response function) to obtain 2000 × 1000 *RR*s for each population, respectively. We averaged the variability stage to obtain 1000 population-level *RR*s, and further calculated 1000 population-level *PAF* and *PIF*. We further performed 1000 iterations for other uncertainties (i.e., base line pediatric asthma incidence, directs cost, and the treatment and hospitalization days) to obtain new pediatric asthma cases attributable to NO_2_ and corresponding economic loss or benefits, and reported their mean and 95% UI, i.e., the 2·5th–97·5th percentile of their values in the 1000 uncertainty runs. Finally, we tested the robustness of the model by performing 250 times of Monte Carlo simulations and calculating the error of those 250 simulations. The result showed that the error was within 5%,^20^ indicating 2000 × 1000 runs were sufficient to quantify the uncertainty of the projected results.

### Role of funding source

The funding source of the study had no role in the study design, data collection, data analysis, data interpretation, or writing of the manuscript. The corresponding author had full access to all the data in the study and had final responsibility for the decision to submit for publication.

## Results

### The pediatric asthma incidence attributable to NO_2_ exposure in 2019

**Figure 2:**
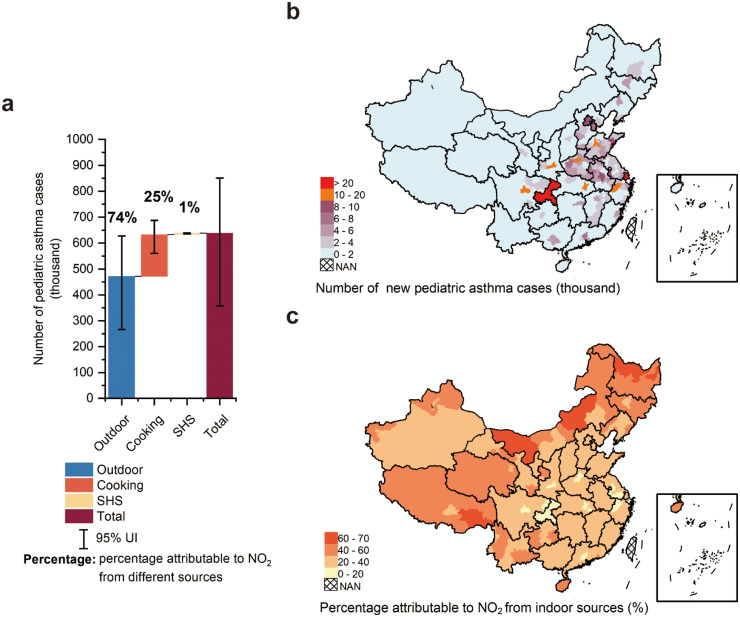
Estimates of number of new pediatric asthma cases attributable to nitrogen dioxide (NO_2_) in urban areas in 2019 (a) number of new pediatric asthma cases from different sources of NO_2_ in 2019 (b) number of new pediatric asthma cases in 330 Chinese cities, and (c) percentage of NO_2_-attributable pediatric asthma incidence from indoor sources to total NO_2_-attributable pediatric asthma incidence in 330 Chinese cities. SHS, second hand smoke.

Long-term exposure to NO_2_ is associated with 637 thousand (95% UI 358–851) new pediatric asthma cases in Chinese urban areas in 2019, equating to 360 (202–482) per 100 000 children (less than 18 years old). NO_2_ from indoor and outdoor sources is associated with 166 thousand (91–223) and 471 thousand (266–628) new pediatric asthma cases in urban areas in China in 2019, respectively, accounting for 74% and 26% of pediatric asthma cases attributable to NO_2_ exposure (Figure 2a). Cooking is the most important indoor source of NO_2_ in urban areas, accounting for 25% (161 thousand (88–216) cases) of pediatric asthma cases attributable to NO_2_ exposure.

Regionally, the larger burden of new pediatric asthma cases associated with NO_2_ is found in areas with higher population density, i.e., cities in eastern China, especially municipalities and provincial capitals (Figure 2b). Shanghai (a municipality), Chongqing (a municipality), and Zhengzhou (the capital of Henan province) are the top three cities with the greatest number of NO_2_-attributable pediatric asthma cases, with 26·1 thousand (15·1–36·8), 24·2 thousand (13·8–35·6) and 14·1 thousand (9·1–18·4), respectively. Figure 2**c** shows the percentage of NO_2_-attributable pediatric asthma incidence from indoor sources to total NO_2_-attributable pediatric asthma incidence (hereinafter referred to “the percentage of contribution from indoor sources”) in 330 Chinese cities. The percentage of contribution from indoor sources is higher in cities in the north and northwest of China, where people open their windows less frequently and spend less time outdoors. The top three cities with the highest percentage of contribution from indoor sources are Hegang (67%), Yichun (65%), and Heihe (64%) in Heilongjiang province.

In general, the incidence of pediatric asthma is highest in the youngest age group and decreases with age, and it is higher in boys than girls. Our estimation support this point (**Table S10** in appendix), with the largest number of new asthma cases attributable to NO_2_ exposure in the youngest age group (less than six years old, 386 thousand (220–521) cases) and a higher number of new cases for boys (385 thousand (212–522) cases) than girls (252 thousand (142–388) cases). However, the number of new asthma cases attributable to NO_2_ exposure is not significantly different between the age groups 7–11 years old and 12–17 years old (7–11 years old, 128 thousand (72–171) cases; 12–17 years old, 123 thousand (62–167) cases). The percentage of contribution from indoor sources is higher for children aged 12–17 years old (31%) than other age groups (less than six years old, 25%; 7–11 years old, 26%), and it is higher for boys (28%) than girls (26%).

### The health benefits of policies on restricting NO_2_ emissions

**Figure 3:**
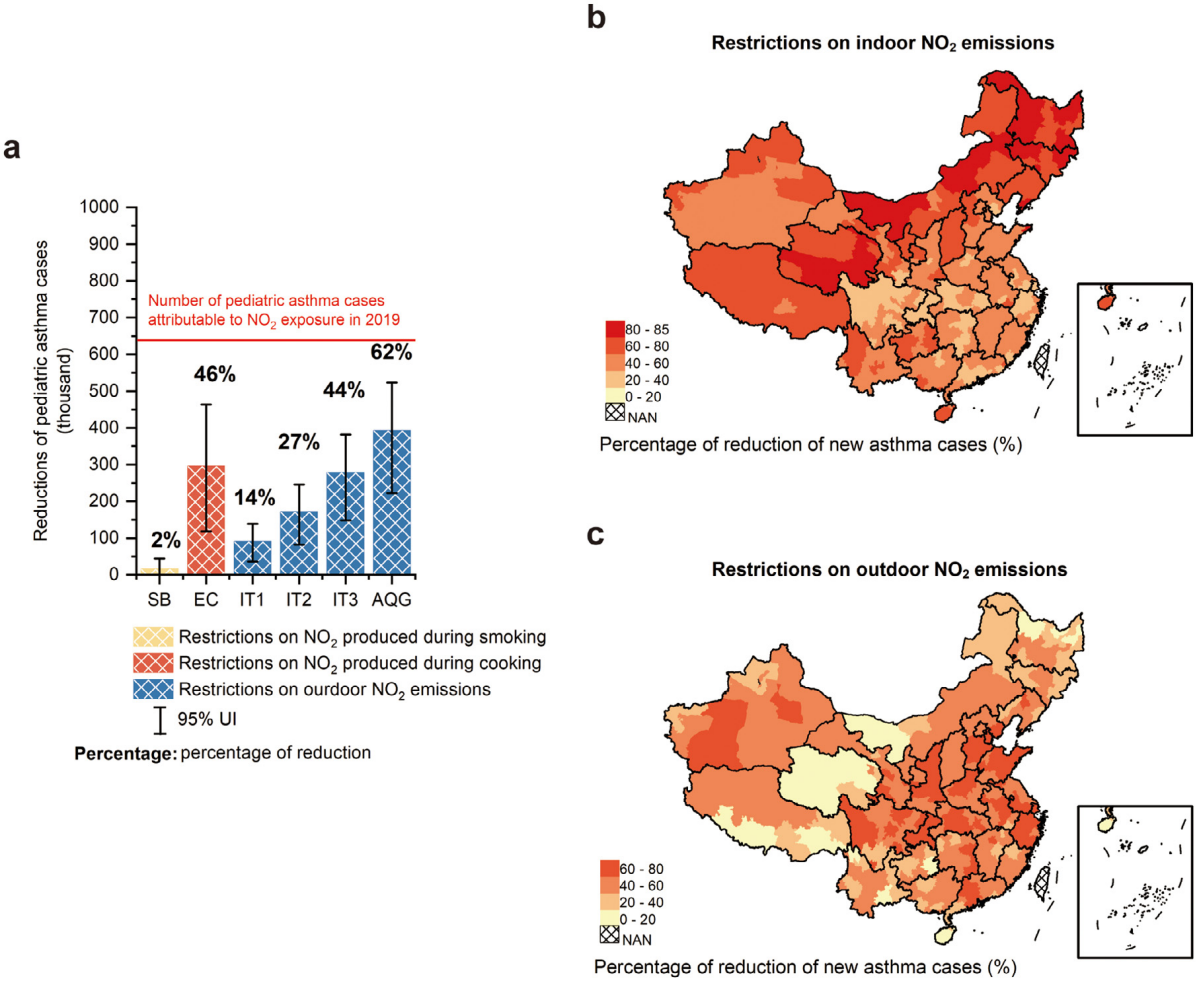
Burden reductions of number of new pediatric asthma cases attributable to NO_2_ by restrictions on NO_2_ emissions. (a) Different restrictions on NO_2_ emissions in China, (b) percentage of reduction of NO_2_-attributable pediatric asthma incidence after restricting indoor NO_2_ emissions by switching from using a gas stove to electric stove for cooking to total NO_2_-attributable pediatric asthma incidence in 330 Chinese cities, (c) percentage of reduction of NO_2_-attributable pediatric asthma incidence after restricting outdoor NO_2_ emissions to meet the World Health Organization Air Quality Guideline for NO_2_ outdoor to total NO_2_-attributable pediatric asthma incidence in 330 Chinese cities. SB, smoking ban, under the condition of no people smoking; EC, switching from using a gas stove to electric stove for cooking, under the condition of all residents using electric stoves for cooking in Chinese urban areas; IT, the outdoor air meet the World Health Organization interim target for NO_2_, IT1 = 40 μg/m^3^, IT2 = 30 μg/m^3^, IT3 = 20 μg/m^3^; AQG, the outdoor air meet the World Health Organization Air Quality Guideline for NO_2_, AQG = 10 μg/m^3^.

The reduction of the burden of new pediatric asthma associated with NO_2_ after restricting the NO_2_ emissions is shown in Figure 3a. Outdoor air meeting the WHO AQG 2021 is projected to be the scenarios with the largest reduction of new pediatric asthma cases, with reductions of 62% (393 thousand (222–523) cases) of new pediatric asthma cases attributable to NO_2_ exposure in 2019. The burden reduction of new pediatric asthma cases by switching from using a gas stove to the electric stove for cooking (EC) is on a par with restricting the NO_2_ emissions in outdoor air to meet WHO interim targets 3 (IT3), with reductions of 46% (296 thousand (119–463) cases) and 44% (278 thousand (148–382) cases) of new pediatric asthma cases attributable to NO_2_ exposure in 2019. The smoking ban has little effect on the number of pediatric asthma cases attributable to NO_2_, with a reduction of 2% (16 thousand (-2–44) cases). The percentage of reduction of NO_2_-attributable pediatric asthma incidence after restricting the indoor and outdoor NO_2_ emissions to total NO_2_-attributable pediatric asthma incidence in 2019 in 330 Chinese cities are shown in Figure 3b and 3c, respectively. Pediatric asthma incidences are estimated to be reduced effectively by restrictions on outdoor NO_2_ emissions in cities with high population density, such as cities in eastern China. While restrictions on indoor NO_2_ emissions effectively reduce pediatric asthma incidences in most areas of China, especially in cities in northern China with more than 80% reductions of new pediatric asthma cases attributable to NO_2_ exposure.

### The economic losses associated with NO_2_-related pediatric asthma

The economic burden of pediatric asthma is 2130 (1380–2870) RMB for each child with asthma, accounting for about 5% of per capita per capita disposable income in China. The per capita cost of pediatric asthma is higher in cities with high per capita Gross Domestic Product, such as Shenzhen (4380 (2630–5960) RMB), Beijing (3620 (2220–4940) RMB), and Shanghai (3510 (2150–4740) RMB) (**Figure S4**). Pediatric asthma attributable to NO_2_ is estimated to cause 1358 million (674–2145) RMB economic losses in Chinese urban areas in 2019. Shanghai was the city with the highest economic losses at 92·7 million (45·1–155·1) RMB. NO_2_ from cooking, second-hand smoking, and outdoor source are associated with 323 million (161–513) RMB, 11 million (6–17) RMB, and 1024 million (505–1617) RMB economic losses (Table 1). The restrictions on indoor and outdoor NO_2_ emissions in urban China would reduce the economic loss from pediatric asthma by up to 594 million (228–1052) and 867 million (427–1364) RMB, respectively (Table 1). Shanghai would be the city with the most reductions of economic loss from pediatric asthma under both restrictions on indoor and outdoor NO_2_ emissions.

**Table 1:** The economic losses due to pediatric asthma attributable to NO_2_ in Chinese urban areas [mean (95% UI) in million RMB].

Sources of NO_2_		In 2019	Reductions after restrictions on NO_2_ emissions	
**Outdoor**		1024 (505, 1617)	**IT1**^a^	209 (79, 365)
			**IT2**	398 (177, 663)
			**IT3**	629 (297, 1011)
			**AQG**^b^	867 (427, 1364)
**Indoor**	**Cooking**	323 (161, 513)	**EC**^c^	594 (228, 1052)
	**Smoking**	11 (6, 17)	**SB**^d^	42 (1, 114)
**Total**		1358 (674, 2145)		

a IT, the outdoor air meets the World Health Organization interim target for NO_2_, IT1 = 40 μg/m^3^, IT2 = 30 μg/m^3^, IT3 = 20 μg/m^3^;

b AQG, the outdoor air meets the World Health Organization Air Quality Guideline for NO_2_, AQG = 10 μg/m^3^;

c EC, using electric stoves for cooking, under the condition of all residents using electric stoves for cooking in Chinese urban areas;

d SB, smoking ban, under the condition of no people smoking.

## Discussion

Our results show that a large number of pediatric asthma cases in Chinese urban areas in 2019 were attributable to NO_2_, with significant contributions from both indoor and outdoor sources. The cost of each child with asthma is equivalent to about 5% of per capita disposable income in China. The reduction of pediatric asthma cases and its corresponding economic losses by restricting indoor NO_2_ emissions is comparable to the burden reduction when outdoor air meets WHO AQG 2021 (i.e., the annual NO_2_ concentration lower than 10 μg/m^3^). The restrictions on indoor and outdoor NO_2_ emissions are of the essence in reducing pediatric asthma incidence in urban China. The unique approach of this study is estimating the disease burden attributable to exposure to NO_2_ from indoor and outdoor sources separately, and comparing the health benefits of restricting indoor and outdoor NO_2_ emissions.

NO_2_ is an oxidizing gas associated with oxidative stress in asthma morbidity. Studies have identified associations between NO_2_ and increased airway responsiveness, airway inflammation, and enhanced responses to allergen.^21^ Two latest studies have shown NO_2_-attributable new pediatric asthma cases at 4·0 million in 2015^6^ and 1·9 million in 2019^22^ globally. The incidence of new pediatric asthma associated with NO_2_ exposure in China in both studies (260 (120–340) per 100 000 children in 2015 and about 129 per 100,000 children in 2019, respectively) are lower than that in our study (360 (202-482) per 100 000 children per year), but similar to the scenario when indoor sources of NO_2_ were completely restricted in our study (194 (109–260) per 100 000 children per year). The biases between our results and those of previous studies reflects the serious impact of NO_2_ from indoor source on the assessment of NO_2_-attributable asthma incidence in children.

*Air Pollution Prevention and Control Action Plan* was implemented in China in 2013, aiming to reduce the concentration of ambient air pollutants. But the concentration of ambient NO_2_ has not decreased significantly in recent years, with the annual average concentration only reduced by 5% from 29·3  g m^−3^ in 2015 to 27·7 μg m^−3^ in 2019.^23^ Ambient NO_2_ is traffic-related air pollution associated with the combustion of gasoline and diesel in vehicles. Transportation contributes up to 80% of ambient NO_2_ in urban areas. Population growth, extensive urbanization, and wealth creation have combined to create ever-increasing traffic volumes in urban areas in China, making it increasingly difficult to reduce ambient NO_2_ pollution. Studies have shown that replacing the traditional fossil energy vehicles with new energy vehicles would lead to substantial reductions of ambient NO_2_ (30–80%) in most of China.^24^ General Office of the State Council of the People's Republic of China issued *the Development Plan for New Energy Vehicle Industry (2021–2035)* on November 2, 2020, to increase the new energy vehicles quotas in total vehicle production, aiming at peaking fossil fuels consumption of land transport by 2030.^25^ This policy would bring benefits in terms of reduce ambient NO_2_ concentrations in China.

Despite the policies on developing new energy vehicles, it might be decades before the ambient NO_2_ meet the WHO AQG 2021. Prior to this time point, restricting NO_2_ from indoor sources would reap health benefits efficiently, especially in reducing pediatric asthma incidence in Chinese urban areas. Switching from using a gas stove to the electric stove for cooking to reduce the NO_2_ produced by gas combustion is a vital strategy to restrict NO_2_ emissions indoors. With the popularity of induction cookers and ceramic cooktops in China in recent years, the acceptability of electric stoves in Chinese families has been increasing year by year. However, using gas stoves for cooking is still mainstream in households in Chinese urban areas.^26^ It is necessary to encourage the use of electric stoves for cooking in Chinese urban areas. The government may need to provide some preferential policies to increase the utilization rate of electric stoves. The increase in the utilization rate of electric stoves is accompanied by the increase in demand for electricity and a decrease in demand for gas in residence. The urban energy system needs to be adjusted accordingly to meet the new demand for energy.

The measures for restriction on NO_2_ emissions from indoor and outdoor reflect the demand to adjust the energy consumption structure of residences and transportations in Chinese urban areas, respectively. Traditional energy consumption is accompanied by greenhouse gas emissions. China pledged to achieve the goal of cutting greenhouse gas emissions to net-zero by 2050 at the 2019 Climate Action Summit.^27^ Developing new energy and constructing new electrical power systems, including the power systems for transportation and residences, are important measures to achieve this goal.^28^ These actions may be a win-win opportunity for not only China but also other developing countries which are facing both climate change and air pollution challenges.

Several limitations exist in our approach. First, we applied the concentration-response function from a meta-analysis of studies in multiple countries around the world^4^ to estimate the NO_2_-attributable burden of pediatric asthma incidence in urban areas in China. The relative risk from east Asia weighted 22·8% in the meta-analysis. Our sensitivity analysis using the pooled relative risk from studies in east Asia showed a low uncertainty (**Figure S3** in appendix). It should be noted that NO_2_ from indoor cooking is mixed with other air pollutants from burning oil, heating ingredient and stirring ingredient. Applying the coefficient of concentration-response function from ambient NO_2_ for the indoor part may introduce some uncertainties. However, NO_2_ is produced from gas combustion during cooking and it can be reduced by changing the type of energy to cook (i.e., switching from gas to electricity), and this is different from those pollutants produced from burning oil, heating ingredient or stirring ingredient. Therefore, other pollutants produced during cooking are not necessarily accompanied by the emission of NO_2_. As our aim was to analyze the disease burden attributable to NO_2_ itself, rather than the disease burden associated with all the air pollutants from cooking, the concentration-response function from ambient NO_2_ is still applicable to this study. Secondly, we estimated exposure to NO_2_ from two main indoor sources, i.e., cooking and second-hand smoke. Another indoor source of NO_2_, wall-mounted gas boiler, was not considered in this study. The wall-mounted gas boilers are equipped with enclosed combustors and exhaust ducts, which emit negligible NO_2_ indoors compared to cooking and smoking. Due to central heating systems in northern China, the wall-mounted gas boilers were mainly used in families in southern China. Studies have shown the low usage of wall-mounted gas boilers in south China.^29^ So the contribution of wall-mounted gas boiler to NO_2_ exposure is too low to affect the incidence of NO_2_-attributable pediatric asthma incidence in this study. Thirdly, we estimated the incidence of pediatric asthma associated with an annual average exposure of NO_2_, while the time period of changes in NO_2_ exposure may also influence the incidence and introduce uncertainties. Zhu et al. studied temporal variations of short-term associations between NO_2_ concentrations and emergency department visits in Shanghai, China, in 2008–2019. They found the associations of emergency department visits and NO_2_ remained stable over the study period.^30^ This evidence means that effects on pediatric asthma incidence for NO_2_ may also remain stable over time, and we expect more direct evidence in future studies.

Despite these limitations, our results showed that indoor and outdoor NO_2_ emissions are associated with the disease burden of pediatric asthma incidence in households without using solid fuels and kerosene. The implementation of the policies on developing clean and alternative energy vehicles has brought hope for reducing ambient NO_2_ pollution in the future. We call on individuals to use the electricity for cooking instead of gas to reduce exposure to NO_2_ from indoor sources. The government may need to adjust the energy structure of residences to meet the new demand for energy. These measurements on restrictions of NO_2_ emissions, i.e., developing new energy vehicles and switching from gas stove to electric stove, support clean energy consumption and aid in achieving climate change mitigation.

## Contributors

**Ying Hu** designed the study and planned the analysis, performed the model analysis, analyzed the results, interpreted the results, validated and completed all figures, and drafted the manuscript. **John S. Ji** drafted and commented on the manuscript. **Bin Zhao** coordinated and supervised the project, designed the study and planned the analysis, analyzed the results, interpreted the results, and drafted the manuscript.

## Data sharing statement

Datasets generated and/or analyzed in the present study are available from the corresponding author upon reasonable request.

Editor note: The Lancet Group takes a neutral position with respect to territorial claims in published maps and institutional affiliations.

## Declaration of interests

The authors declare no competing interests.

## Appendix Supplementary materials

Notes S1-2, Figures S1-4, Tables S1-10. Image, application 1

Annual number of new asthma cases per 100 000 children attributable to nitrogen dioxide before and after restrictions on NO2 emissions in 330 Chinese cities. Image, application 2
