# Supplementary material for: Restrictions on indoor and outdoor NO2 emissions to reduce disease burden for pediatric asthma in China: A modeling study
Source: Lancet Reg Health West Pac. 2022 May 4;24:100463. doi: 10.1016/j.lanwpc.2022.100463 (PMC9079688; doi:10.1016/j.lanwpc.2022.100463)
Supplement: Supplementary file 1 — Notes S1-2, Figures S1-4, Tables S1-10. [file mmc1.docx]

**Supplementary material for “Restrictions on indoor and outdoor NO_2_ emissions to reduce disease burden for pediatric asthma in China: a modeling study”**

**Table of Contents**

Note S1. Source-specific model to estimate human exposure to NO_2_ from indoor and outdoor sources 2

Note S2. The proportion of the population exposed to second-hand smoke. 3

Figure S1 The validation of the source-specific exposure model. ^1^ 4

Figure S2 The proportion of contribution of different sources in NO_2_ exposure in 330 Chinese cities. 5

Figure S3 Sensitivity analysis using the pooled relative risk according to studies from east Asia. 6

Figure S4 Economic losses in urban areas in 330 Chinese cities. 7

Table S1 The concentration of human exposure to NO_2_ from outdoor sources (*C_ambient_*), cooking (*C_cooking_*), and second-hand smoke (*C_SHS_*) and the proportion of the population exposed to second-hand smoke (*P_SHS_*) after restrictions on NO_2_ emissions. 8

Table S2 The population and per capita Gross Domestic Product per day in urban areas of 330 Chinese city in 2019. 9

Table S3 The exposure factor of NO_2_ in 31 provinces in China. 12

Table S4 The incidence of pediatric asthma in China in 2019 13

Table S5 Percentages for populations with different ages and genders in different provinces and municipalities 14

Table S6 Per capita direct cost and per capita treatment and hospitalization days 15

Table S7 Smoking rates in China in 2019 16

Table S8 Percentages for different number of occupants in 31 provinces. 17

Table S9 The average relative risk in urban areas in China. 18

Table S10 NO_2_-attribuatable pediatric asthma cases for children of different age and gender. 19

Dataset S1. Annual number of new asthma cases per 100 000 children attributable to nitrogen dioxide before and after restrictions on NO_2_ emissions in 330 Chinese cities (Excel file).

# Note S1. Source-specific model to estimate human exposure to NO_2_ from indoor and outdoor sources

First, we calculated the concentration of NO_2_ indoor from indoor and outdoor sources using the mass balance equations

$$\begin{aligned} \frac{{dC}_{in,i}}{dt}=-\left( a+k_{s} \right)C_{in,i}+\frac{S}{V}\#\left( S1 \right) \end{aligned}$$

and

$$\begin{aligned} \frac{{dC}_{in,o}}{dt}=-\left( a+k_{s} \right)C_{in,o}+aC_{out}\#\left( S2 \right) \end{aligned}$$

where *C_in,i_* is the concentration of indoor-originated NO_2_ in indoor environment (μg m^−3^), *C_in,o_* is the concentration of outdoor-originated NO_2_ in indoor environment (μg m^−3^), *C_out_* is the outdoor concentration of the NO_2_ (μg m^−3^), *k_s_* is the removal rate for indoor surfaces through heterogeneous reactions between NO_2_ and indoor surfaces (h^−1^), *a* is the air exchange rate (h^−1^), depending on ventilation of the room (opening and closing windows, and operating a range hood). *S* is the air pollutant emission rate (μg h^−1^) including NO_2_ emissions during cooking and smoking in this study, *V* is the room volume (m^3^).

Secondly, we calculated the concentration of NO_2_ exposure from indoor and outdoor sources during studied time period (time period *t_1_* to *t_2_*). They were calculated based on the concentration, activity time and respiratory rate in microenvironments:

$$\begin{aligned} C_{exp,i}\left( t_{1},t_{2} \right)=\frac{\int_{t_{1}}^{t_{2}} C_{s,i}Qdt}{\int_{t_{1}}^{t_{2}} Qdt}\#\left( S3 \right) \end{aligned}$$

and

$$\begin{aligned} C_{exp,o}\left( t_{1},t_{2} \right)=\frac{\int_{t_{1}}^{t_{2}} C_{s,o}Qdt}{\int_{t_{1}}^{t_{2}} Qdt},\#\left( S4 \right) \end{aligned}$$

where *C_exp,i_* the concentration of human exposure to NO_2_ from indoor sources (μg m^−3^), *C_exp,o_* the concentration of human exposure to NO_2_ from outdoor sources (μg m^−3^), *C_s,i_* is the concentration of the indoor-originated NO_2_ in microenvironment *s* in which people are doing activities (μg m^−3^), *C_s,o_* is the concentration of the outdoor-originated NO_2_ in microenvironment *s* in which people are doing activities (μg m^−3^), and *Q* is the respiratory rate during the activity (m^3^ h^−1^).

In this model, we used NO_2_ concentrations in residences as proxies for concentrations in all non-outdoor settings as inadequate information was available to simulate NO_2_ exposure in non-outdoor environments such as offices, schools, shops, and vehicles. It will not introduce unacceptable errors to estimates of exposure to NO_2_ because most people spend about 69% of their lives in their residences, which is much more than they spend in other non-outdoor environments, and cooking and second-hand smoking in residences are the most important indoor sources of NO_2_. The microenvironment *s* in this study, could be a kitchen, living room or bedroom, or the outdoor environment. When the door between two room in opened (i.e. the door between kitchen and living room), the air in the two rooms is considered to be well mixed. Ventilation, indoor sources, human activities and other parameters related to people’s living habits were obtained by a questionnaire survey in our previous study.^1^ The value of input parameters *k_s_*, *a*, *S*, *V*, *Q* and *C_out_* were shown in that study. The validation of the model was also provided in that study by comparing the modeled results with measured results in previous studies under the same condition (**Figure S1**).

# Note S2. The proportion of the population exposed to second-hand smoke.

When someone is smoking indoors, the NO_2_ produced by tobacco combustion is released into the room, and all people (including the person who is smoking) in the room are exposed to these NO_2_. We used the proportion of the population exposed to second-hand smoke to represent the proportion of the population exposed to NO_2_ produced by tobacco combustion, which was calculated by

$$\begin{aligned} P_{SHS,g}=1-\left( 1-P_{smoking,g} \right)\left( \sum_{f} P_{number,f}\left( 1-P_{smoking} \right)^{f-1} \right)\#\left( S5 \right) \end{aligned}$$

where *P_SHS_* is the proportion of the population exposed to second-hand smoke and the subscript *g* represents the population with specific age and gender. *P_smoking_* is the current smoking rate for people of all ages and genders, *P_smoking,g_* is the current smoking rate for people with specific age and gender *g*. *P_number,f_* is the proportion of the population that the family household size is *f*. The interpretation of **Equation (S5)** is as follows: $\left( 1-P_{smoking,g} \right)$ is the proportion of the target population do not smoke, and $\left( \sum_{f} P_{number,f}\left( 1-P_{smoking} \right)^{f-1} \right)$ is the proportion of the other family members of the target population’s family do not smoke, so $\left( 1-P_{smoking,g} \right)\left( \sum_{f} P_{number,f}\left( 1-P_{smoking} \right)^{f-1} \right)$ is the proportion of the all person of the target population’s family do not smoke and $1-\left( 1-P_{smoking,g} \right)\left( \sum_{f} P_{number,f}\left( 1-P_{smoking} \right)^{f-1} \right)$ is the proportion of at least one person of the target population’s family smoke. The age-, sex-, and provincial-specific smoking rates in 2019 (**Table S7**) were calculated using the age-, sex-, and provincial-specific smoking rates in 2013^2^ and the ratio of smoking rates between 2013^2^ and 2019^3^. The proportion of family household size 1 to 10 in 31 Chinese provinces (**Table S8**) were obtained from the National Bureau of Statistics of China^4^.
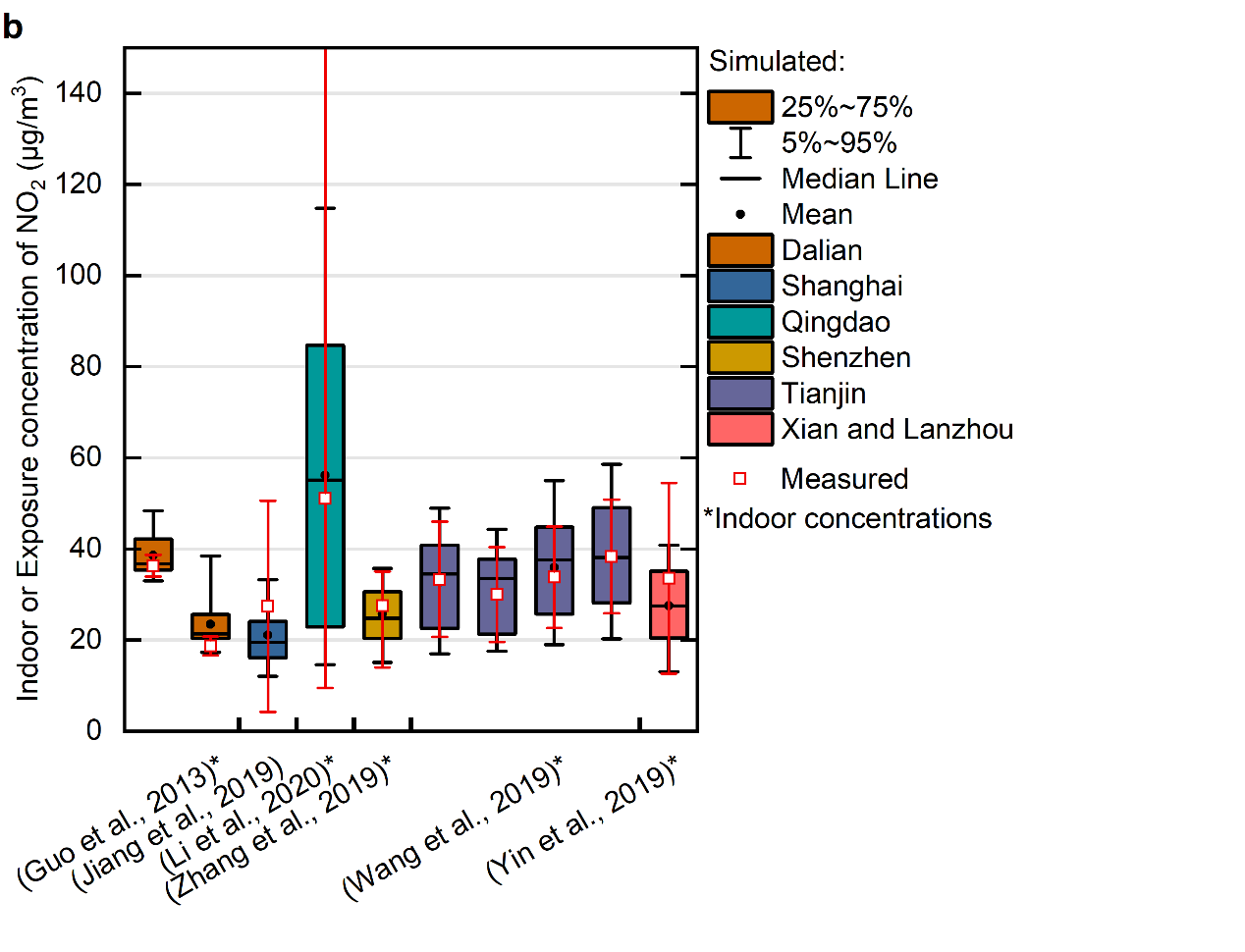


**Figure S1 The validation of the source-specific exposure model.** ^1^


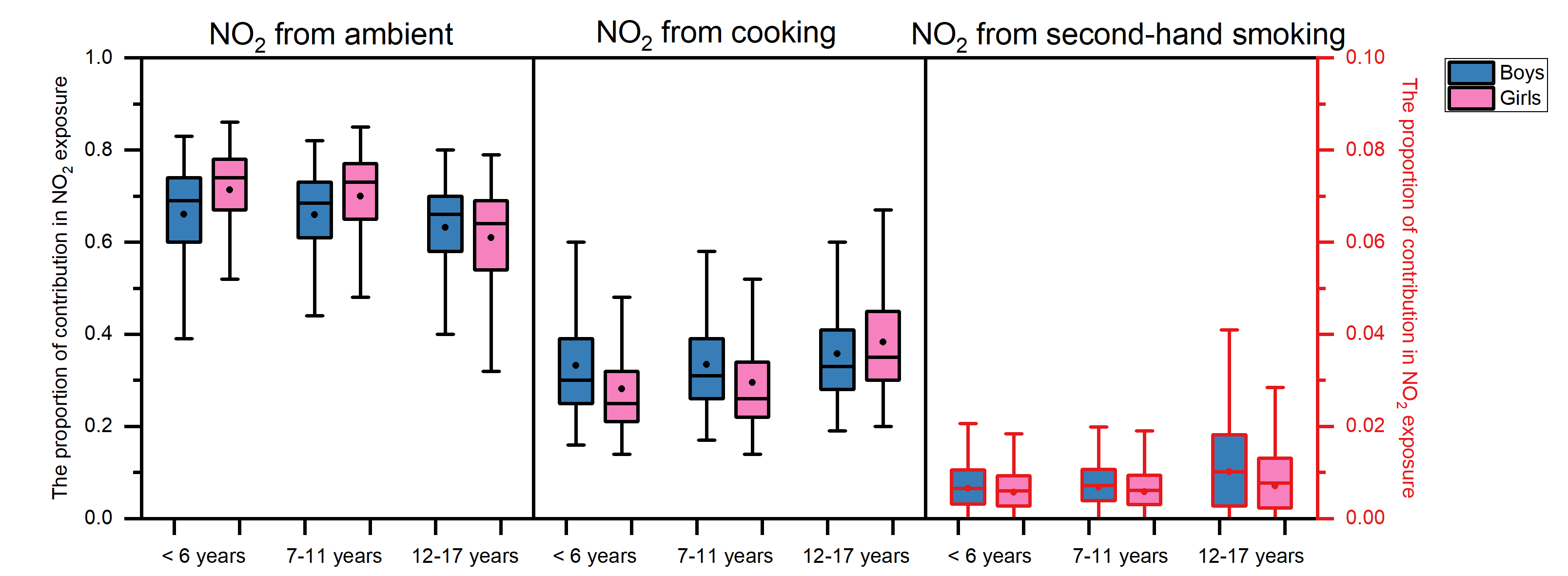


**Figure S2 The proportion of contribution of different sources in NO_2_ exposure in 330 Chinese cities.**

Black line, the proportion of contribution from ambient or cooking in NO_2_ exposure; Red line, the proportion of contribution from second-hand smoking in NO_2_ exposure.


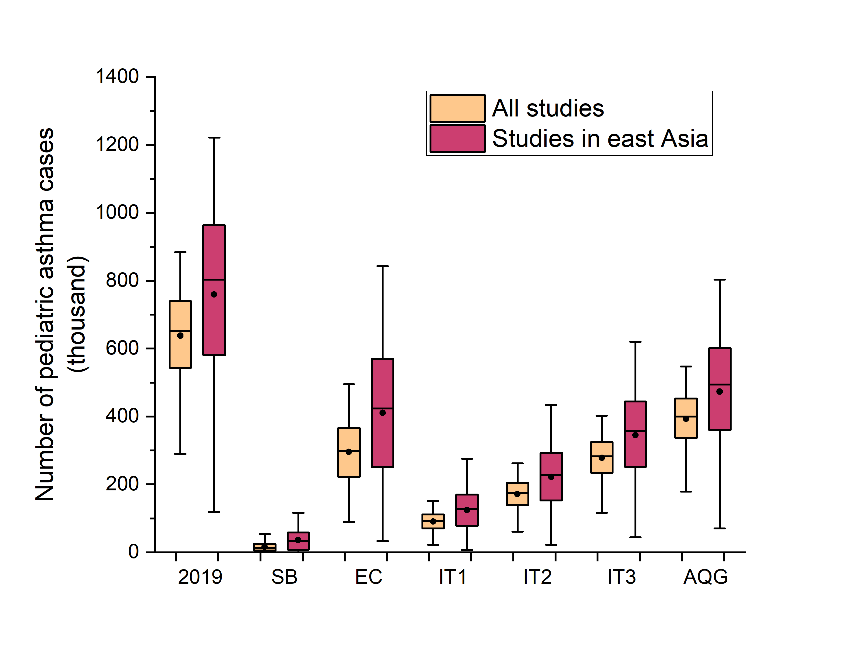


**Figure S3 Sensitivity analysis using the pooled relative risk according to studies from east Asia.**

SB, smoking ban, under the condition of no people smoking; EC, switching from using a gas stove to electric stove for cooking, under the condition of all residents using electric stoves for cooking in Chinese urban areas; IT, the outdoor air meet the World Health Organization interim target for NO_2_, IT1 = 40 μg/m^3^, IT2 = 30 μg/m^3^, IT3 = 20 μg/m^3^; AQG, the outdoor air meet the World Health Organization Air Quality Guideline for NO_2_, AQG = 10 μg/m^3^.

**
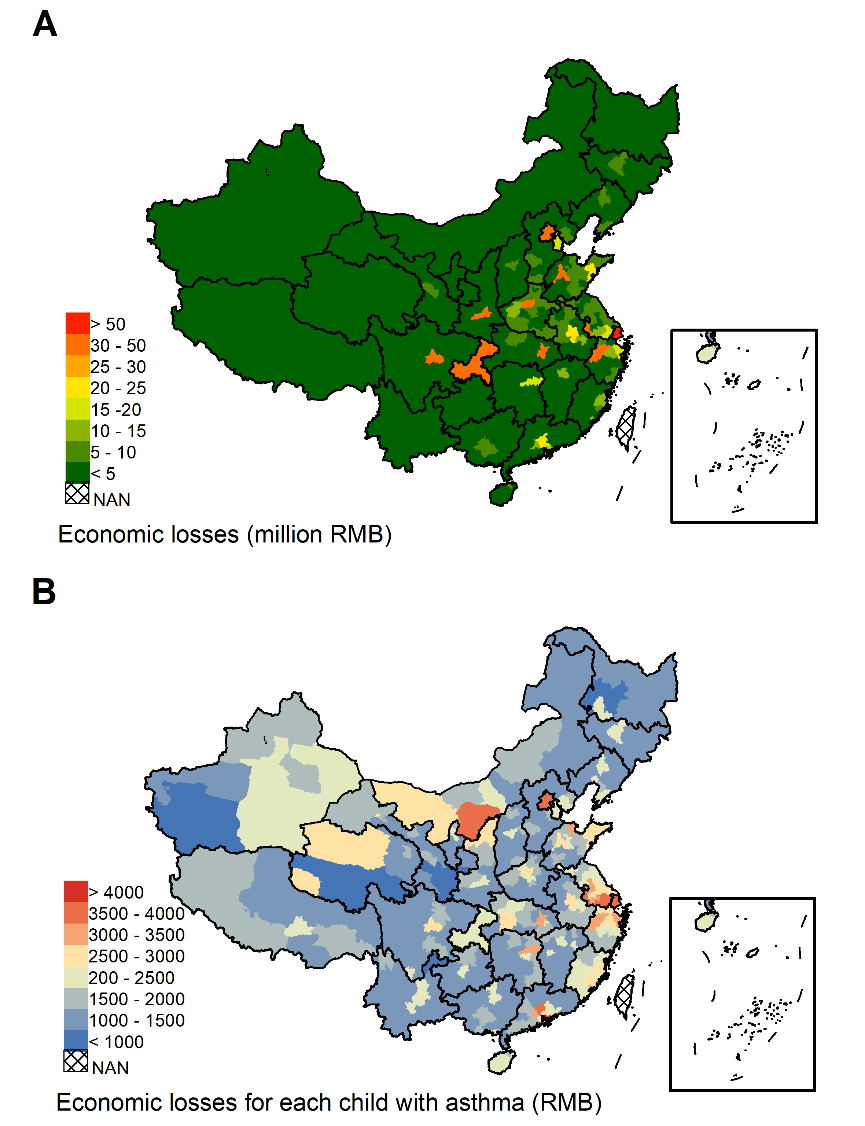
**

**Figure S4 Economic losses in urban areas in 330 Chinese cities. A, total economic losses; B, Economic for each child.**

**Table S1 The concentration of human exposure to NO_2_ from outdoor sources (*C_ambient_*), cooking (*C_cooking_*), and second-hand smoke (*C_SHS_*) and the proportion of the population exposed to second-hand smoke (*P_SHS_*) after restrictions on NO_2_ emissions.**

| **Restrictions on NO_2_ emissions** | ***C_ambient_*** | ***C_cooking_*** | ***C_SHS_*** | ***P_SHS_*** |
| --- | --- | --- | --- | --- |
| SB^a^ | *C_ambient_* in 2019 | *C_cooking_* in 2019 | — — | 0 |
| EC^b^ | *C_ambient_* in 2019 | 0 | *C_SHS_* in 2019 | *P_SHS_* in 2019 |
| IT1^c^ | Equation (5), *Target*=40μg/m^3^ | *C_cooking_* in 2019 | *C_SHS_* in 2019 | *P_SHS_* in 2019 |
| IT2 | Equation (5), *Target*=30μg/m^3^ | *C_cooking_* in 2019 | *C_SHS_* in 2019 | *P_SHS_* in 2019 |
| IT3 | Equation (5), *Target*=20μg/m^3^ | *C_cooking_* in 2019 | *C_SHS_* in 2019 | *P_SHS_* in 2019 |
| AQG^d^ | Equation (5), *Target*=10μg/m^3^ | *C_cooking_* in 2019 | *C_SHS_* in 2019 | *P_SHS_* in 2019 |

^a^ SB, smoking ban, under the condition of no people smoking;

^b^ EC, using electric stoves for cooking, under the condition of all residents using electric stoves for cooking in Chinese urban areas;

^c^ IT, the outdoor air meets the World Health Organization interim target for NO_2_, IT1 = 40 μg/m^3^, IT2 = 30 μg/m^3^, IT3 = 20 μg/m^3^;

^d^ AQG, the outdoor air meets the World Health Organization Air Quality Guideline for NO_2_, AQG = 10 μg/m^3^.

**Table S2 The population (unit: thousand) and per capita Gross Domestic Product per day (GDPp, unit: RMB) in urban areas of 330 Chinese city in 2019.**

| **City** | **Province** | **Population** | **GDPp** | **City** | **Province** | **Population** | **GDPp** | **City** | **Province** | **Population** | **GDPp** |
| --- | --- | --- | --- | --- | --- | --- | --- | --- | --- | --- | --- |
| Beijing | Beijing | 18654 | 450 | Wuhu | Anhui | 2477 | 263 | Guilin | Guangxi | 1870 | 113 |
| Tianjin | Tianjin | 13040 | 248 | Suzhou | Anhui | 3166 | 95 | Hechi | Guangxi | 1423 | 68 |
| Baoding | Hebei | 5916 | 87 | Xuancheng | Anhui | 1411 | 161 | Hezhou | Guangxi | 1716 | 92 |
| Cangzhou | Hebei | 1208 | 131 | Fuzhou | Fujian | 6115 | 331 | Laibin | Guangxi | 1605 | 80 |
| Chengde | Hebei | 1228 | 113 | Longyan | Fujian | 2256 | 278 | Liuzhou | Guangxi | 2582 | 211 |
| Handan | Hebei | 7820 | 100 | Nanping | Fujian | 1813 | 203 | Nanning | Guangxi | 5554 | 169 |
| Hengshui | Hebei | 2088 | 92 | Ningde | Fujian | 1096 | 231 | Qinzhou | Guangxi | 2149 | 112 |
| Langfang | Hebei | 1801 | 179 | Putian | Fujian | 5187 | 245 | Wuzhou | Guangxi | 1130 | 89 |
| Qinhuangdao | Hebei | 3009 | 141 | Quanzhou | Fujian | 2488 | 313 | Yulin | Guangxi | 1605 | 78 |
| Shijianzhuang | Hebei | 8741 | 145 | Sanming | Fujian | 611 | 276 | Haikou | Hainan | 4164 | 198 |
| Tangshan | Hebei | 6858 | 237 | Xiamen | Fujian | 5503 | 391 | Sanyan | Hainan | 1433 | 239 |
| Xingtai | Hebei | 1883 | 79 | Zhangzhou | Fujian | 1349 | 252 | Chongqing | Chongqing | 20868 | 208 |
| Zhangjiakou | Hebei | 3193 | 96 | Fuzhou | Jiangxi | 3040 | 102 | Chengdu | Sichuan | 11501 | 283 |
| Datong | Shanxi | 3245 | 104 | Ganzhou | Jiangxi | 4082 | 110 | Dazhou | Sichuan | 2311 | 98 |
| Jincheng | Shanxi | 811 | 158 | Jian | Jiangxi | 1060 | 115 | Deyang | Sichuan | 1234 | 180 |
| Jinzhong | Shanxi | 1298 | 118 | Jingdezhen | Jiangxi | 848 | 151 | Ganmei | Sichuan | 512 | 89 |
| Linfen | Shanxi | 1663 | 88 | Jiujiang | Jiangxi | 1803 | 174 | Guangan | Sichuan | 1654 | 106 |
| Lvliang | Shanxi | 588 | 107 | Nanchang | Jiangxi | 5549 | 275 | Guangyuan | Sichuan | 1208 | 97 |
| Suzhou | Shanxi | 1379 | 163 | Pingxiang | Jiangxi | 1555 | 132 | Leshan | Sichuan | 1523 | 156 |
| Taiyuan | Shanxi | 6084 | 248 | Shangrao | Jiangxi | 4047 | 101 | Liangshan | Sichuan | 2363 | 93 |
| Xinzhou | Shanxi | 1115 | 87 | Xinyu | Jiangxi | 1608 | 224 | Luzhou | Sichuan | 1996 | 132 |
| Yangquan | Shanxi | 1399 | 139 | Yichun | Jiangxi | 2068 | 132 | Meishan | Sichuan | 1575 | 126 |
| Yuncheng | Shanxi | 1440 | 80 | Yingtan | Jiangxi | 1131 | 219 | Mianyang | Sichuan | 2297 | 161 |
| Changzhi | Shanxi | 3184 | 129 | Binzhou | Shandong | 1784 | 172 | Nanchong | Sichuan | 2547 | 99 |
| Alxa League | Inner Mongolia | 376 | 324 | Dezhou | Shandong | 2025 | 143 | Neijiang | Sichuan | 1825 | 106 |
| Bayan Nur | Inner Mongolia | 978 | 142 | Dongying | Shandong | 1816 | 367 | Panzhihua | Sichuan | 853 | 226 |
| Baitou | Inner Mongolia | 2954 | 257 | Heze | Shandong | 3809 | 106 | Suining | Sichuan | 1917 | 115 |
| Chifeng | Inner Mongolia | 2408 | 108 | Jinan | Shandong | 11170 | 292 | Yanan | Sichuan | 814 | 129 |
| Ordos | Inner Mongolia | 583 | 474 | Jining | Shandong | 3070 | 143 | Yinbin | Sichuan | 3020 | 156 |
| Hohhot | Inner Mongolia | 2653 | 244 | Laiwu | Shandong | 0 | 0 | Ziyang | Sichuan | 1405 | 85 |
| Hulun Buir | Inner Mongolia | 696 | 129 | Liaocheng | Shandong | 3005 | 102 | Zigong | Sichuan | 1930 | 134 |
| Tongliao | Inner Mongolia | 1580 | 111 | Linyi | Shandong | 4597 | 118 | Bijie | Guizhou | 2185 | 91 |
| Wuhai | Inner Mongolia | 828 | 267 | Qingdao | Shandong | 8502 | 340 | Guiyang | Guizhou | 3452 | 225 |
| Ulanqab | Inner Mongolia | 602 | 106 | Rizhao | Shandong | 2250 | 182 | Liupanshui | Guizhou | 646 | 118 |
| Xilingol League | Inner Mongolia | 1336 | 207 | Taian | Shandong | 2780 | 129 | Qiandongnan | Guizhou | 2327 | 126 |
| Hinggan League | Inner Mongolia | 1110 | 89 | Weihai | Shandong | 2202 | 287 | Qiannan | Guizhou | 2133 | 87 |
| Anshan | Liaoning | 2046 | 135 | Weifang | Shandong | 3150 | 166 | Tongren | Guizhou | 659 | 108 |
| Benxi | Liaoning | 1233 | 128 | Yantai | Shandong | 3118 | 294 | Zunyi | Guizhou | 2935 | 152 |
| Chaoyang | Liaoning | 855 | 79 | Zaozhuang | Shandong | 4002 | 118 | Baoshan | Yunnan | 1425 | 100 |
| Dalian | Liaoning | 5676 | 274 | Zibo | Shandong | 4661 | 212 | Chuxiong | Yunnan | 1638 | 125 |
| Dandong | Liaoning | 1079 | 88 | Anyang | Henan | 2506 | 118 | Dali | Yunnan | 2168 | 104 |
| Fushun | Liaoning | 1892 | 113 | Hebi | Henan | 1369 | 166 | Dehong | Yunnan | 970 | 107 |
| Fuxin | Liaoning | 1037 | 77 | Jiaozuo | Henan | 2064 | 210 | Diqing | Yunnan | 182 | 169 |
| Huludao | Liaoning | 1359 | 87 | Kaifeng | Henan | 3622 | 142 | Honghe | Yunnan | 3245 | 127 |
| Jinzhou | Liaoning | 1331 | 97 | Luoyang | Henan | 4401 | 200 | Kunming | Yunnan | 4928 | 257 |
| Liaoyang | Liaoning | 1191 | 125 | Luohe | Henan | 2843 | 162 | Lijiang | Yunnan | 243 | 100 |
| Panjin | Liaoning | 1444 | 244 | Nanyang | Henan | 4296 | 104 | Lincang | Yunnan | 500 | 82 |
| Shenyang | Liaoning | 8591 | 213 | Pingdingshan | Henan | 2338 | 129 | Nujiang | Yunnan | 440 | 95 |
| Tieling | Liaoning | 589 | 67 | Puyang | Henan | 1579 | 120 | Puer | Yunnan | 364 | 91 |
| Yingkou | Liaoning | 1317 | 149 | Sanxiamen | Henan | 1327 | 174 | Qujing | Yunnan | 2153 | 117 |
| Baicheng | Jilin | 697 | 71 | Shangqiu | Henan | 3980 | 109 | Wenshan | Yunnan | 1971 | 81 |
| Baishan | Jilin | 755 | 120 | Xinxiang | Henan | 2317 | 138 | Xishuangbannan | Yunnan | 925 | 131 |
| Jilin | Jilin | 2614 | 94 | Xinyang | Henan | 3327 | 117 | Yuxi | Yunnan | 1137 | 224 |
| Liaoyuan | Jilin | 653 | 96 | Xuchang | Henan | 2843 | 209 | Zhaotong | Yunnan | 1456 | 58 |
| Siping | Jilin | 973 | 68 | Zhengzhou | Henan | 8361 | 310 | Ali | Tibet | 66 | 152 |
| Songyuan | Jilin | 813 | 73 | Zhoukou | Henan | 1348 | 101 | Changdu | Tibet | 157 | 76 |
| Tonghua | Jilin | 624 | 92 | Zhumadian | Henan | 1811 | 107 | Lhasa | Tibet | 407 | 238 |
| Yanbian | Jilin | 2091 | 95 | Ezhou | Hubei | 1889 | 292 | Linzhi | Tibet | 66 | 201 |
| Changchun | Jilin | 6462 | 215 | Enshi | Hubei | 2614 | 94 | Naqu | Tibet | 144 | 83 |
| Daqing | Heilongjiang | 2206 | 258 | Huanggang | Hubei | 590 | 101 | Rikaze | Tibet | 171 | 189 |
| Da Hinggan Ling | Heilongjiang | 580 | 84 | Huangshi | Hubei | 1046 | 196 | Shannan | Tibet | 92 | 136 |
| Haerbin | Heilongjiang | 8906 | 133 | Jingmen | Hubei | 1096 | 192 | Ankang | Shaanxi | 1490 | 121 |
| Hegang | Heilongjiang | 966 | 93 | Jingzhou | Hubei | 1822 | 124 | Baiji | Shaanxi | 2030 | 162 |
| Heihe | Heilongjiang | 290 | 100 | Shiyan | Hubei | 2007 | 162 | Hanzhong | Shaanxi | 1665 | 123 |
| Jixi | Heilongjiang | 1224 | 88 | Suizhou | Hubei | 1113 | 144 | Shangluo | Shaanxi | 818 | 96 |
| Jiamusi | Heilongjiang | 1224 | 90 | Wuhan | Hubei | 15282 | 399 | Tongchuan | Shaanxi | 1022 | 123 |
| Mudanjiang | Heilongjiang | 1385 | 90 | Xianning | Hubei | 1063 | 172 | Weinan | Shaanxi | 1388 | 94 |
| Qitaihe | Heilongjiang | 757 | 82 | Xiangyang | Hubei | 3846 | 232 | Xian | Shaanxi | 11992 | 253 |
| Qiqihar | Heilongjiang | 2110 | 62 | Xiaogan | Hubei | 1619 | 128 | Xianyang | Shaanxi | 803 | 138 |
| Shuangyashan | Heilongjiang | 741 | 93 | Yichang | Hubei | 2159 | 295 | Yanan | Shaanxi | 979 | 202 |
| Suihua | Heilongjiang | 1305 | 58 | Changde | Hunan | 3450 | 171 | Yulin | Shaanxi | 847 | 331 |
| Yichun | Heilongjiang | 1143 | 72 | Chenzhou | Hunan | 1957 | 139 | Baiyin | Gansu | 679 | 77 |
| Shanghai | Shanghai | 21439 | 431 | Hengyang | Hunan | 2471 | 127 | Dingxi | Gansu | 638 | 40 |
| Changzhou | Jiangsu | 4715 | 428 | Huaihua | Hunan | 979 | 89 | Gannan | Gansu | 367 | 83 |
| Huaian | Jiangsu | 5147 | 215 | Loudi | Hunan | 1517 | 114 | Jiayuguan | Gansu | 407 | 307 |
| Lianyungang | Jiangsu | 3467 | 190 | Shaoyang | Hunan | 1688 | 80 | Jinchang | Gansu | 285 | 201 |
| Nanjing | Jiangsu | 10940 | 454 | Xiangtan | Hunan | 2104 | 215 | Jingquan | Gansu | 557 | 150 |
| Nantong | Jiangsu | 3313 | 351 | Xiangxi | Hunan | 3083 | 73 | Lanzhou | Gansu | 2879 | 206 |
| Suzhou | Jiangsu | 5778 | 491 | Yiyang | Hunan | 3303 | 111 | Linxia | Gansu | 1046 | 40 |
| Taizhou | Jiangsu | 2527 | 303 | Yongzhou | Hunan | 2862 | 101 | Longnan | Gansu | 815 | 46 |
| Wuxi | Jiangsu | 4130 | 493 | Yueyang | Hunan | 2716 | 179 | Pingliang | Gansu | 720 | 59 |
| Suqian | Jiangsu | 2743 | 172 | Zhangjiajie | Hunan | 1321 | 98 | Qingyang | Gansu | 543 | 90 |
| Xuzhou | Jiangsu | 5285 | 222 | Changsha | Hunan | 8905 | 383 | Tianshui | Gansu | 1793 | 52 |
| Yancheng | Jiangsu | 3760 | 217 | Zhuzhou | Hunan | 3229 | 204 | Wuwei | Gansu | 1413 | 73 |
| Yangzhou | Jiangsu | 3590 | 353 | Chaozhou | Guangdong | 2450 | 111 | Zhangye | Gansu | 706 | 99 |
| Zhenjiang | Jiangsu | 1587 | 353 | Dongguan | Guangdong | 11305 | 308 | Guoluo | Qinghai | 100 | 60 |
| Hangzhou | Zhejiang | 13293 | 418 | Foshan | Guangdong | 6682 | 367 | Haibei | Qinghai | 150 | 88 |
| Huzhou | Zhejiang | 2266 | 281 | Guangzhou | Guangdong | 13827 | 429 | Hainan | Qinghai | 226 | 100 |
| Jainxing | Zhejiang | 1902 | 309 | Heyuan | Guangdong | 464 | 95 | Hiaxi | Qinghai | 451 | 351 |
| Jinhua | Zhejiang | 2023 | 223 | Huizhou | Guangdong | 2479 | 236 | Huangnan | Qinghai | 138 | 99 |
| Lishui | Zhejiang | 850 | 183 | Jiangmen | Guangdong | 2116 | 187 | Xining | Qinghai | 1267 | 153 |
| Ningbo | Zhejiang | 6090 | 392 | Jieyang | Guangdong | 3073 | 94 | Yushuo | Qinghai | 276 | 39 |
| Quzhou | Zhejiang | 1720 | 195 | Maoming | Guangdong | 4421 | 140 | Guyuan | Ningxia | 642 | 71 |
| Shaoxing | Zhejiang | 4532 | 314 | Meizhou | Guangdong | 1406 | 74 | Shizuishan | Ningxia | 614 | 174 |
| Taizhou | Zhejiang | 3298 | 229 | Qingyuan | Guangdong | 2160 | 120 | Wuzhong | Ningxia | 572 | 112 |
| Wenzhou | Zhejiang | 3520 | 195 | Shantou | Guangdong | 8175 | 131 | Yinchuan | Ningxia | 1745 | 229 |
| Panshan | Zhejiang | 1457 | 320 | Shanwei | Guangdong | 754 | 99 | Zhongwei | Ningxia | 586 | 102 |
| Anqing | Anhui | 1214 | 139 | Shaoguan | Guangdong | 1333 | 120 | Aksu | Xinjiang | 1181 | 117 |
| Bangfu | Anhui | 1903 | 166 | Shenzhen | Guangdong | 7986 | 558 | Altay | Xinjiang | 349 | 141 |
| Haozhou | Anhui | 2788 | 91 | Yangjiang | Guangdong | 1797 | 138 | Bozhou | Xinjiang | 282 | 203 |
| Chizhou | Anhui | 1099 | 154 | Yunfu | Guangdong | 1000 | 100 | Changji | Xinjiang | 832 | 226 |
| Chuzhou | Anhui | 919 | 193 | Zhanjiang | Guangdong | 2464 | 114 | Hami | Xinjiang | 577 | 269 |
| Fuyang | Anhui | 3805 | 90 | Fuqing | Guangdong | 2116 | 148 | Hetian | Xinjiang | 738 | 41 |
| Hefei | Anhui | 4773 | 317 | Zhongshan | Guangdong | 4334 | 254 | Kashi | Xinjiang | 1436 | 62 |
| Huaibei | Anhui | 1722 | 131 | Zhuhai | Guangdong | 1928 | 481 | Karamay | Xinjiang | 416 | 52 |
| Huainan | Anhui | 3002 | 102 | Baise | Guangxi | 516 | 94 | Kezhou | Xinjiang | 215 | 70 |
| Huangshan | Anhui | 771 | 159 | Beihai | Guangxi | 977 | 211 | Tacheng | Xinjiang | 523 | 148 |
| Liuan | Anhui | 3641 | 91 | Chongzuo | Guangxi | 530 | 99 | Turopan | Xinjiang | 389 | 167 |
| Maanshan | Anhui | 1345 | 246 | Fangchenggang | Guangxi | 823 | 200 | Urumqi | Xinjiang | 2979 | 265 |
| Tongling | Anhui | 1493 | 161 | Guigang | Guangxi | 2861 | 78 | Ili Kazak | Xinjiang | 1718 | 117 |

**Table S3 The exposure factor of NO_2_ in 31 provinces in China.**

|  | **Mean** | **SD** | **P5** | **P25** | **P50** | **P75** | **P95** |
| --- | --- | --- | --- | --- | --- | --- | --- |
| Beijing | 0.50 | 0.15 | 0.28 | 0.39 | 0.48 | 0.60 | 0.79 |
| Tianjin | 0.47 | 0.15 | 0.26 | 0.37 | 0.45 | 0.56 | 0.77 |
| Hebei | 0.46 | 0.14 | 0.26 | 0.36 | 0.45 | 0.55 | 0.72 |
| Shanxi | 0.45 | 0.13 | 0.25 | 0.36 | 0.44 | 0.53 | 0.69 |
| Inner Mongolia | 0.45 | 0.13 | 0.25 | 0.35 | 0.43 | 0.53 | 0.69 |
| Liaoning | 0.42 | 0.15 | 0.20 | 0.31 | 0.40 | 0.51 | 0.69 |
| Jilin | 0.40 | 0.14 | 0.19 | 0.30 | 0.39 | 0.49 | 0.66 |
| Heilongjiang | 0.41 | 0.15 | 0.18 | 0.30 | 0.40 | 0.51 | 0.69 |
| Shanghai | 0.49 | 0.14 | 0.29 | 0.39 | 0.48 | 0.58 | 0.76 |
| Jiangsu | 0.47 | 0.13 | 0.27 | 0.37 | 0.45 | 0.55 | 0.71 |
| Zhejiang | 0.50 | 0.15 | 0.29 | 0.39 | 0.48 | 0.59 | 0.78 |
| Anhui | 0.48 | 0.14 | 0.28 | 0.38 | 0.47 | 0.57 | 0.75 |
| Fujian | 0.58 | 0.17 | 0.32 | 0.45 | 0.57 | 0.70 | 0.86 |
| Jiangxi | 0.57 | 0.16 | 0.32 | 0.44 | 0.56 | 0.69 | 0.87 |
| Shandong | 0.47 | 0.16 | 0.25 | 0.35 | 0.45 | 0.57 | 0.79 |
| Henan | 0.51 | 0.16 | 0.27 | 0.39 | 0.49 | 0.61 | 0.81 |
| Hubei | 0.52 | 0.14 | 0.32 | 0.42 | 0.51 | 0.61 | 0.77 |
| Hunan | 0.54 | 0.15 | 0.32 | 0.43 | 0.53 | 0.64 | 0.82 |
| Guangdong | 0.60 | 0.15 | 0.37 | 0.49 | 0.59 | 0.71 | 0.86 |
| Guangxi | 0.54 | 0.14 | 0.33 | 0.44 | 0.53 | 0.63 | 0.81 |
| Hainan | 0.62 | 0.15 | 0.38 | 0.51 | 0.62 | 0.74 | 0.88 |
| Chongqing | 0.56 | 0.14 | 0.36 | 0.46 | 0.55 | 0.65 | 0.82 |
| Sichuan | 0.54 | 0.14 | 0.33 | 0.44 | 0.52 | 0.62 | 0.80 |
| Guizhou | 0.51 | 0.15 | 0.29 | 0.40 | 0.49 | 0.60 | 0.80 |
| Yunnan | 0.53 | 0.13 | 0.33 | 0.43 | 0.52 | 0.61 | 0.76 |
| Tibet | 0.47 | 0.13 | 0.28 | 0.38 | 0.46 | 0.55 | 0.70 |
| Shaanxi | 0.48 | 0.13 | 0.29 | 0.39 | 0.47 | 0.56 | 0.73 |
| Gansu | 0.49 | 0.14 | 0.28 | 0.39 | 0.47 | 0.57 | 0.74 |
| Qinghai | 0.47 | 0.13 | 0.27 | 0.37 | 0.45 | 0.54 | 0.71 |
| Ningxia | 0.50 | 0.16 | 0.27 | 0.39 | 0.48 | 0.59 | 0.80 |
| Xinjiang | 0.48 | 0.13 | 0.27 | 0.38 | 0.47 | 0.55 | 0.71 |

**Table S4 The incidence of pediatric asthma in China in 2019** (per 100 000 children, mean (95%CI))

| **Age groups** | **1-4 years** | | **5-9 years** | | **10-14 years** | | **15-19 years** | |
| --- | --- | --- | --- | --- | --- | --- | --- | --- |
| **Gender** | **Males** | **Females** | **Males** | **Females** | **Males** | **Females** | **Males** | **Females** |
| Beijing | 2120 (1179, 3515) | 1563 (860, 2588) | 949 (450, 1636) | 659 (306, 1149) | 470 (190, 791) | 425 (174, 725) | 300 (169, 471) | 286 (157, 452) |
| Tianjin | 1779 (989, 2950) | 1312 (722, 2172) | 797 (377, 1373) | 553 (257, 964) | 395 (159, 664) | 357 (146, 609) | 251 (142, 395) | 240 (132, 380) |
| Hebei | 1022 (569, 1695) | 754 (415, 1248) | 458 (217, 789) | 318 (148, 554) | 227 (92, 382) | 205 (84, 350) | 144 (82, 227) | 138 (76, 218) |
| Shanxi | 1014 (564, 1682) | 748 (411, 1238) | 454 (215, 783) | 315 (146, 550) | 225 (91, 379) | 203 (83, 347) | 143 (81, 225) | 137 (75, 216) |
| Inner Mongolia | 748 (416, 1240) | 552 (304, 913) | 335 (159, 577) | 233 (108, 405) | 166 (67, 279) | 150 (61, 256) | 106 (60, 166) | 101 (56, 160) |
| Liaoning | 1347 (749, 2233) | 993 (546, 1644) | 603 (286, 1039) | 419 (194, 730) | 299 (121, 503) | 270 (110, 461) | 190 (108, 299) | 181 (100, 287) |
| Jilin | 1297 (721, 2150) | 956 (526, 1583) | 581 (275, 1001) | 403 (187, 703) | 288 (116, 484) | 260 (106, 444) | 183 (104, 288) | 175 (96, 277) |
| Heilongjiang | 856 (476, 1420) | 631 (347, 1045) | 383 (182, 661) | 266 (124, 464) | 190 (77, 320) | 172 (70, 293) | 121 (68, 190) | 115 (64, 183) |
| Shanghai | 4763 (2649, 7898) | 3512 (1932, 5816) | 2133 (1010, 3676) | 1480 (687, 2581) | 1057 (427, 1778) | 955 (391, 1630) | 673 (381, 1058) | 642 (354, 1017) |
| Jiangsu | 2003 (1114, 3322) | 1477 (813, 2446) | 897 (425, 1546) | 623 (289, 1086) | 444 (179, 748) | 402 (164, 686) | 283 (160, 445) | 270 (149, 428) |
| Zhejiang | 2540 (1412, 4211) | 1873 (1030, 3101) | 1137 (539, 1960) | 789 (366, 1376) | 563 (227, 948) | 509 (208, 869) | 359 (203, 564) | 342 (189, 542) |
| Anhui | 3387 (1884, 5617) | 2498 (1374, 4136) | 1517 (718, 2614) | 1053 (489, 1836) | 751 (303, 1265) | 679 (278, 1159) | 479 (271, 753) | 457 (252, 723) |
| Fujian | 2581 (1436, 4280) | 1903 (1047, 3151) | 1156 (547, 1992) | 802 (372, 1399) | 573 (231, 964) | 517 (212, 883) | 365 (206, 574) | 348 (192, 551) |
| Jiangxi | 1937 (1077, 3211) | 1428 (786, 2365) | 867 (411, 1495) | 602 (279, 1050) | 430 (173, 723) | 388 (159, 663) | 274 (155, 430) | 261 (144, 413) |
| Shandong | 2226 (1238, 3690) | 1641 (903, 2718) | 997 (472, 1718) | 692 (321, 1206) | 494 (199, 831) | 446 (183, 762) | 315 (178, 495) | 300 (165, 475) |
| Henan | 2652 (1475, 4397) | 1955 (1076, 3238) | 1187 (562, 2046) | 824 (383, 1437) | 588 (237, 990) | 532 (218, 907) | 375 (212, 589) | 357 (197, 566) |
| Hubei | 2012 (1119, 3335) | 1483 (816, 2456) | 901 (427, 1552) | 625 (290, 1090) | 446 (180, 751) | 403 (165, 688) | 284 (161, 447) | 271 (149, 429) |
| Hunan | 1496 (832, 2481) | 1103 (607, 1827) | 670 (317, 1155) | 465 (216, 811) | 332 (134, 559) | 300 (123, 512) | 211 (120, 333) | 202 (111, 319) |
| Guangdong | 1133 (630, 1879) | 836 (460, 1384) | 507 (240, 875) | 352 (163, 614) | 251 (101, 423) | 227 (93, 388) | 160 (91, 252) | 153 (84, 242) |
| Guangxi | 1671 (929, 2770) | 1232 (678, 2040) | 748 (354, 1289) | 519 (241, 905) | 371 (150, 624) | 335 (137, 572) | 236 (134, 371) | 225 (124, 357) |
| Hainan | 2411 (1341, 3997) | 1778 (978, 2943) | 1079 (511, 1860) | 749 (348, 1306) | 535 (216, 900) | 483 (198, 825) | 341 (193, 536) | 325 (179, 514) |
| Chongqing | 3026 (1683, 5017) | 2231 (1227, 3694) | 1355 (642, 2335) | 940 (437, 1640) | 671 (271, 1130) | 607 (248, 1035) | 428 (242, 672) | 408 (225, 646) |
| Sichuan | 2843 (1581, 4714) | 2096 (1153, 3471) | 1273 (603, 2194) | 884 (410, 1541) | 631 (255, 1061) | 570 (233, 973) | 402 (227, 632) | 383 (211, 607) |
| Guizhou | 1147 (638, 1902) | 846 (465, 1401) | 514 (243, 885) | 357 (165, 622) | 254 (103, 428) | 230 (94, 393) | 162 (92, 255) | 155 (85, 245) |
| Yunnan | 648 (361, 1075) | 478 (263, 792) | 290 (138, 500) | 202 (94, 351) | 144 (58, 242) | 130 (53, 222) | 92 (52, 144) | 87 (48, 138) |
| Tibet | 349 (194, 579) | 257 (142, 426) | 156 (74, 269) | 109 (50, 189) | 77 (31, 130) | 70 (29, 119) | 49 (28, 78) | 47 (26, 75) |
| Shaanxi | 2573 (1431, 4266) | 1897 (1044, 3141) | 1152 (546, 1986) | 800 (371, 1394) | 571 (230, 961) | 516 (211, 880) | 364 (206, 572) | 347 (191, 549) |
| Gansu | 1987 (1105, 3294) | 1465 (806, 2426) | 890 (421, 1533) | 617 (287, 1077) | 441 (178, 742) | 398 (163, 680) | 281 (159, 441) | 268 (148, 424) |
| Qinghai | 840 (467, 1392) | 619 (341, 1025) | 376 (178, 648) | 261 (121, 455) | 186 (75, 313) | 168 (69, 287) | 119 (67, 187) | 113 (62, 179) |
| Ningxia | 549 (305, 910) | 405 (223, 670) | 246 (116, 423) | 171 (79, 297) | 122 (49, 205) | 110 (45, 188) | 78 (44, 122) | 74 (41, 117) |
| Xinjiang | 840 (467, 1392) | 619 (341, 1025) | 376 (178, 648) | 261 (121, 455) | 186 (75, 313) | 168 (69, 287) | 119 (67, 187) | 113 (62, 179) |

**Table S5 Percentages for populations with different ages and genders in different provinces and municipalities (%). (Values for a province are used for all the cities in that province.)**

| **Age groups**  **(years old)** | **0 - 0.5** | | **0.5 - 1** | | **1 - 2** | | **3- 6** | | **7 - 11** | | **12 - 17** | |
| --- | --- | --- | --- | --- | --- | --- | --- | --- | --- | --- | --- | --- |
| **Gender** | **Male** | **Female** | **Male** | **Female** | **Male** | **Female** | **Male** | **Female** | **Male** | **Female** | **Male** | **Female** |
| Beijing | 0.15 | 0.14 | 0.54 | 0.48 | 1.15 | 1.03 | 0.27 | 0.24 | 1.62 | 1.44 | 2.53 | 2.27 |
| Tianjin | 0.17 | 0.15 | 0.56 | 0.49 | 1.17 | 1.03 | 0.33 | 0.29 | 1.98 | 1.74 | 2.97 | 2.64 |
| Hebei | 0.33 | 0.29 | 1.06 | 0.91 | 2.19 | 1.88 | 0.60 | 0.52 | 3.38 | 2.94 | 3.71 | 3.45 |
| Shanxi | 0.25 | 0.23 | 0.80 | 0.72 | 1.63 | 1.47 | 0.55 | 0.50 | 3.60 | 3.29 | 4.98 | 4.74 |
| Inner Mongolia | 0.22 | 0.19 | 0.70 | 0.64 | 1.46 | 1.32 | 0.46 | 0.42 | 2.92 | 2.67 | 3.69 | 3.43 |
| Liaoning | 0.16 | 0.14 | 0.54 | 0.49 | 1.15 | 1.04 | 0.39 | 0.35 | 2.46 | 2.20 | 3.09 | 2.85 |
| Jilin | 0.17 | 0.15 | 0.60 | 0.54 | 1.30 | 1.16 | 0.42 | 0.38 | 2.53 | 2.28 | 3.17 | 2.96 |
| Heilongjiang | 0.17 | 0.15 | 0.56 | 0.50 | 1.16 | 1.05 | 0.41 | 0.37 | 2.56 | 2.34 | 3.18 | 3.01 |
| Shanghai | 0.15 | 0.13 | 0.53 | 0.47 | 1.16 | 1.01 | 0.30 | 0.25 | 1.69 | 1.47 | 2.27 | 2.11 |
| Jiangsu | 0.23 | 0.20 | 0.78 | 0.65 | 1.63 | 1.35 | 0.45 | 0.37 | 2.68 | 2.22 | 3.58 | 3.18 |
| Zhejiang | 0.22 | 0.19 | 0.71 | 0.61 | 1.47 | 1.28 | 0.46 | 0.41 | 2.79 | 2.46 | 3.45 | 3.13 |
| Anhui | 0.35 | 0.27 | 1.04 | 0.82 | 2.07 | 1.64 | 0.62 | 0.50 | 3.79 | 3.08 | 4.45 | 3.92 |
| Fujian | 0.30 | 0.24 | 0.95 | 0.76 | 1.95 | 1.56 | 0.55 | 0.46 | 3.21 | 2.69 | 3.95 | 3.43 |
| Jiangxi | 0.36 | 0.29 | 1.28 | 0.99 | 2.76 | 2.09 | 0.82 | 0.63 | 4.84 | 3.72 | 4.78 | 3.96 |
| Shandong | 0.28 | 0.23 | 0.91 | 0.74 | 1.88 | 1.53 | 0.56 | 0.48 | 3.29 | 2.85 | 3.38 | 3.03 |
| Henan | 0.30 | 0.25 | 1.20 | 0.97 | 2.70 | 2.13 | 0.78 | 0.60 | 4.56 | 3.57 | 4.64 | 4.02 |
| Hubei | 0.29 | 0.23 | 0.86 | 0.70 | 1.73 | 1.40 | 0.48 | 0.38 | 2.89 | 2.32 | 4.16 | 3.52 |
| Hunan | 0.33 | 0.27 | 1.04 | 0.86 | 2.12 | 1.75 | 0.66 | 0.54 | 3.78 | 3.11 | 3.70 | 3.28 |
| Guangdong | 0.28 | 0.24 | 0.88 | 0.72 | 1.80 | 1.47 | 0.55 | 0.44 | 3.64 | 2.94 | 5.13 | 4.53 |
| Guangxi | 0.43 | 0.35 | 1.26 | 1.04 | 2.50 | 2.08 | 0.78 | 0.66 | 4.60 | 3.91 | 4.61 | 3.97 |
| Hainan | 0.39 | 0.31 | 1.16 | 0.93 | 2.32 | 1.86 | 0.69 | 0.54 | 4.28 | 3.32 | 5.12 | 4.27 |
| Chongqing | 0.24 | 0.21 | 0.84 | 0.75 | 1.80 | 1.60 | 0.57 | 0.50 | 3.59 | 3.17 | 4.34 | 4.07 |
| Sichuan | 0.24 | 0.22 | 0.82 | 0.74 | 1.73 | 1.56 | 0.56 | 0.50 | 3.59 | 3.20 | 4.37 | 4.06 |
| Guizhou | 0.38 | 0.31 | 1.16 | 0.94 | 2.35 | 1.89 | 0.85 | 0.73 | 5.59 | 4.87 | 5.81 | 5.28 |
| Yunnan | 0.32 | 0.29 | 1.00 | 0.89 | 2.04 | 1.81 | 0.72 | 0.64 | 4.49 | 3.99 | 4.91 | 4.45 |
| Tibet | 0.39 | 0.37 | 1.25 | 1.18 | 2.57 | 2.45 | 0.81 | 0.78 | 4.91 | 4.73 | 5.34 | 5.02 |
| Shaanxi | 0.24 | 0.21 | 0.77 | 0.66 | 1.59 | 1.36 | 0.49 | 0.41 | 3.13 | 2.61 | 4.59 | 4.00 |
| Gansu | 0.29 | 0.25 | 0.88 | 0.75 | 1.77 | 1.50 | 0.60 | 0.53 | 3.92 | 3.43 | 5.19 | 4.77 |
| Qinghai | 0.34 | 0.30 | 1.02 | 0.93 | 2.06 | 1.90 | 0.71 | 0.66 | 4.38 | 4.11 | 5.00 | 4.72 |
| Ningxia | 0.32 | 0.28 | 1.04 | 0.92 | 2.16 | 1.91 | 0.73 | 0.66 | 4.55 | 4.15 | 5.09 | 4.85 |
| Xinjiang | 0.38 | 0.36 | 1.11 | 1.05 | 2.19 | 2.08 | 0.66 | 0.62 | 4.04 | 3.82 | 4.60 | 4.36 |

**Table S6 Per capita direct cost and per capita treatment and hospitalization days**

| **Age** | **0-2 years old** | **3-6 years old** | **> 7 years old** |
| --- | --- | --- | --- |
| Direct cost, Median (IQR), RMB | 550.40  (194.77, 1454.79) | 475.08  (170.86, 1000.87) | 495.64  (156.96, 1424.10) |
| Treatment and hospitalization days,  Mean (SD), days | 7.15 (2.58) | 7.25 (2.83) | 6.08 (2.11) |

**Table S7 Smoking rates in China in 2019**

| **Gender** | **Male** | | | | | **Female** | | | | | **All** |
| --- | --- | --- | --- | --- | --- | --- | --- | --- | --- | --- | --- |
| **Age groups** | **12 - 17 y** | **18 - 44 y** | **45 - 59 y** | **60 - 79 y** | **≥ 80 y** | **12 - 17 y** | **18 - 44 y** | **45 - 59 y** | **60 - 79 y** | **≥ 80 y** |  |
| Beijing | 0.30 | 0.48 | 0.61 | 0.53 | 0.47 | 0.02 | 0.04 | 0.05 | 0.04 | 0.04 | 0.26 |
| Tianjin | 0.29 | 0.46 | 0.59 | 0.51 | 0.46 | 0.02 | 0.04 | 0.05 | 0.04 | 0.04 | 0.26 |
| Hebei | 0.31 | 0.49 | 0.63 | 0.55 | 0.49 | 0.02 | 0.04 | 0.05 | 0.04 | 0.04 | 0.24 |
| Shanxi | 0.33 | 0.52 | 0.66 | 0.58 | 0.52 | 0.03 | 0.04 | 0.05 | 0.05 | 0.04 | 0.26 |
| Inner Mongolia | 0.34 | 0.54 | 0.69 | 0.60 | 0.54 | 0.03 | 0.04 | 0.06 | 0.05 | 0.04 | 0.28 |
| Liaoning | 0.26 | 0.42 | 0.53 | 0.46 | 0.42 | 0.02 | 0.03 | 0.04 | 0.04 | 0.03 | 0.22 |
| Jilin | 0.32 | 0.51 | 0.65 | 0.57 | 0.51 | 0.03 | 0.04 | 0.05 | 0.05 | 0.04 | 0.27 |
| Heilongjiang | 0.30 | 0.48 | 0.61 | 0.53 | 0.48 | 0.02 | 0.04 | 0.05 | 0.04 | 0.04 | 0.25 |
| Shanghai | 0.25 | 0.39 | 0.50 | 0.43 | 0.39 | 0.02 | 0.03 | 0.04 | 0.03 | 0.03 | 0.21 |
| Jiangsu | 0.29 | 0.46 | 0.59 | 0.51 | 0.46 | 0.02 | 0.04 | 0.05 | 0.04 | 0.04 | 0.23 |
| Zhejiang | 0.28 | 0.44 | 0.57 | 0.49 | 0.44 | 0.02 | 0.04 | 0.05 | 0.04 | 0.04 | 0.23 |
| Anhui | 0.32 | 0.51 | 0.65 | 0.56 | 0.51 | 0.03 | 0.04 | 0.05 | 0.04 | 0.04 | 0.24 |
| Fujian | 0.30 | 0.47 | 0.60 | 0.52 | 0.47 | 0.02 | 0.04 | 0.05 | 0.04 | 0.04 | 0.23 |
| Jiangxi | 0.33 | 0.52 | 0.67 | 0.58 | 0.52 | 0.03 | 0.04 | 0.05 | 0.05 | 0.04 | 0.24 |
| Shandong | 0.27 | 0.43 | 0.55 | 0.47 | 0.43 | 0.02 | 0.03 | 0.04 | 0.04 | 0.03 | 0.21 |
| Henan | 0.31 | 0.50 | 0.63 | 0.55 | 0.50 | 0.03 | 0.04 | 0.05 | 0.04 | 0.04 | 0.23 |
| Hubei | 0.31 | 0.50 | 0.64 | 0.55 | 0.50 | 0.03 | 0.04 | 0.05 | 0.04 | 0.04 | 0.25 |
| Hunan | 0.34 | 0.54 | 0.69 | 0.60 | 0.54 | 0.03 | 0.04 | 0.05 | 0.05 | 0.04 | 0.27 |
| Guangdong | 0.28 | 0.44 | 0.57 | 0.49 | 0.44 | 0.02 | 0.04 | 0.05 | 0.04 | 0.04 | 0.22 |
| Guangxi | 0.31 | 0.50 | 0.63 | 0.55 | 0.49 | 0.03 | 0.04 | 0.05 | 0.04 | 0.04 | 0.23 |
| Hainan | 0.26 | 0.41 | 0.53 | 0.46 | 0.41 | 0.02 | 0.03 | 0.04 | 0.04 | 0.03 | 0.20 |
| Chongqing | 0.35 | 0.56 | 0.71 | 0.62 | 0.55 | 0.03 | 0.04 | 0.06 | 0.05 | 0.04 | 0.27 |
| Sichuan | 0.34 | 0.53 | 0.68 | 0.59 | 0.53 | 0.03 | 0.04 | 0.05 | 0.05 | 0.04 | 0.26 |
| Guizhou | 0.47 | 0.74 | 0.94 | 0.82 | 0.74 | 0.04 | 0.06 | 0.08 | 0.07 | 0.06 | 0.33 |
| Yunnan | 0.39 | 0.62 | 0.79 | 0.69 | 0.62 | 0.03 | 0.05 | 0.06 | 0.05 | 0.05 | 0.30 |
| Tibet | 0.25 | 0.40 | 0.50 | 0.44 | 0.39 | 0.02 | 0.03 | 0.04 | 0.03 | 0.03 | 0.18 |
| Shaanxi | 0.33 | 0.52 | 0.67 | 0.58 | 0.52 | 0.03 | 0.04 | 0.05 | 0.05 | 0.04 | 0.27 |
| Gansu | 0.35 | 0.55 | 0.70 | 0.61 | 0.55 | 0.03 | 0.04 | 0.06 | 0.05 | 0.04 | 0.27 |
| Qinghai | 0.34 | 0.54 | 0.69 | 0.60 | 0.54 | 0.03 | 0.04 | 0.06 | 0.05 | 0.04 | 0.26 |
| Ningxia | 0.31 | 0.49 | 0.62 | 0.54 | 0.48 | 0.02 | 0.04 | 0.05 | 0.04 | 0.04 | 0.23 |
| Xinjiang | 0.21 | 0.33 | 0.42 | 0.36 | 0.33 | 0.02 | 0.03 | 0.03 | 0.03 | 0.03 | 0.16 |

**Table S8 Percentages for different number of occupants in 31 provinces.** (Values for a province are used for all the cities in that province)

|  | **Number of occupants (%)** | | | | | | | | | |
| --- | --- | --- | --- | --- | --- | --- | --- | --- | --- | --- |
|  | **1** | **2** | **3** | **4** | **5** | **6** | **7** | **8** | **9** | **10** |
| Beijing | 24.82 | 30.24 | 29.08 | 9.33 | 4.78 | 1.17 | 0.35 | 0.15 | 0.04 | 0.04 |
| Tianjin | 12.56 | 28.64 | 37.09 | 12.87 | 6.35 | 1.91 | 0.39 | 0.13 | 0.03 | 0.03 |
| Hebei | 9.25 | 21.69 | 26.76 | 21.79 | 11.75 | 5.90 | 1.81 | 0.62 | 0.24 | 0.18 |
| Shanxi (Taiyuan) | 10.75 | 21.89 | 27.82 | 21.87 | 10.73 | 4.83 | 1.35 | 0.43 | 0.18 | 0.13 |
| Inner Mongolia | 11.66 | 29.61 | 36.31 | 14.11 | 5.99 | 1.70 | 0.40 | 0.14 | 0.05 | 0.04 |
| Liaoning | 13.45 | 29.78 | 34.67 | 12.54 | 6.97 | 1.98 | 0.43 | 0.13 | 0.03 | 0.02 |
| Jilin | 12.43 | 27.37 | 33.29 | 13.92 | 8.73 | 2.94 | 0.86 | 0.31 | 0.10 | 0.06 |
| Heilongjiang | 12.88 | 28.85 | 35.11 | 12.58 | 7.44 | 2.20 | 0.60 | 0.22 | 0.06 | 0.04 |
| Shanghai | 19.89 | 33.99 | 30.89 | 8.88 | 5.14 | 0.88 | 0.22 | 0.07 | 0.02 | 0.02 |
| Jiangsu | 14.08 | 28.46 | 28.49 | 14.26 | 10.12 | 3.14 | 0.91 | 0.34 | 0.12 | 0.08 |
| Zhejiang | 20.36 | 31.56 | 26.75 | 12.27 | 6.37 | 1.91 | 0.47 | 0.18 | 0.07 | 0.06 |
| Anhui | 15.95 | 25.04 | 26.11 | 18.07 | 9.30 | 3.68 | 1.13 | 0.43 | 0.17 | 0.12 |
| Fujian | 18.35 | 23.64 | 25.02 | 17.37 | 9.61 | 3.83 | 1.28 | 0.53 | 0.21 | 0.15 |
| Jiangxi | 8.93 | 17.32 | 23.00 | 24.10 | 14.17 | 7.05 | 2.92 | 1.23 | 0.62 | 0.65 |
| Shandong | 11.40 | 26.65 | 32.04 | 17.61 | 8.37 | 2.83 | 0.70 | 0.24 | 0.09 | 0.06 |
| Henan | 10.20 | 19.17 | 23.50 | 23.69 | 13.26 | 6.61 | 2.25 | 0.76 | 0.31 | 0.26 |
| Hubei | 12.30 | 23.37 | 28.51 | 18.27 | 10.94 | 4.32 | 1.34 | 0.57 | 0.22 | 0.15 |
| Hunan | 12.52 | 20.68 | 24.56 | 21.07 | 12.35 | 5.50 | 1.92 | 0.77 | 0.34 | 0.28 |
| Guangdong | 21.88 | 22.61 | 19.70 | 15.43 | 10.21 | 5.08 | 2.50 | 1.25 | 0.61 | 0.73 |
| Guangxi | 14.42 | 20.11 | 22.94 | 20.21 | 12.39 | 5.53 | 2.38 | 1.04 | 0.48 | 0.50 |
| Hainan | 17.34 | 17.13 | 19.44 | 19.92 | 13.44 | 6.61 | 3.13 | 1.42 | 0.72 | 0.85 |
| Chongqing | 22.28 | 27.33 | 24.80 | 14.62 | 7.36 | 2.58 | 0.70 | 0.22 | 0.07 | 0.05 |
| Sichuan | 17.54 | 25.27 | 25.30 | 16.69 | 9.57 | 3.80 | 1.18 | 0.39 | 0.15 | 0.12 |
| Guizhou | 15.41 | 22.68 | 23.41 | 18.86 | 11.40 | 5.12 | 1.97 | 0.70 | 0.26 | 0.19 |
| Yunnan | 11.19 | 17.22 | 22.21 | 23.87 | 14.00 | 7.46 | 2.52 | 0.91 | 0.36 | 0.27 |
| Tibet | 14.70 | 16.19 | 16.22 | 15.20 | 11.34 | 7.87 | 5.97 | 4.34 | 2.98 | 5.19 |
| Shaanxi | 12.59 | 21.80 | 26.36 | 20.36 | 11.68 | 5.14 | 1.40 | 0.42 | 0.15 | 0.09 |
| Gansu | 10.28 | 19.60 | 24.96 | 20.06 | 13.28 | 7.63 | 2.70 | 0.93 | 0.34 | 0.23 |
| Qinghai | 11.92 | 19.12 | 24.78 | 20.44 | 12.34 | 6.61 | 2.62 | 1.10 | 0.53 | 0.55 |
| Ningxia | 12.20 | 23.00 | 28.09 | 19.57 | 10.21 | 4.57 | 1.57 | 0.51 | 0.17 | 0.13 |
| Xinjiang | 17.13 | 21.72 | 25.60 | 17.94 | 10.75 | 3.70 | 1.68 | 0.75 | 0.35 | 0.37 |

**Table S9 The average relative risk in urban areas in China.**

|  | **Parameter in the main text** | **Mean (95%UI)** |  |
| --- | --- | --- | --- |
| In 2019 | $\bar{RR}$^a^ | 1.80 (1.33, 2.45) |  |
| SB | $\bar{{RR}_{SB}}$^b^ | 1.78 (1.33, 2.37) |  |
| EC | $\bar{{RR}_{EC}}$^c^ | 1.43 (1.22, 1.66) |  |
| IT1 | $\bar{{RR}_{IT1}}$^d^ | 1.68 (1.30, 2.21) |  |
| IT2 | $\bar{{RR}_{IT2}}$^e^ | 1.58 (1.25, 2.03) |  |
| IT3 | $\bar{{RR}_{IT3}}$^f^ | 1.45 (1.19, 1.80) |  |
| AQG | $\bar{{RR}_{AQG}}$^g^ | 1.31 (1.13, 1.56) |  |

^a^ the average relative risk in 2019;

^b^ the average relative risk after smoking ban;

^c^ the average relative risk after switching from using a gas stove to electric stove for cooking;

^d^ the average relative risk when the outdoor air meets the WHO interim targets 1;

^e^ the average relative risk when the outdoor air meets the WHO interim targets 2;

^f^ the average relative risk when the outdoor air meets the WHO interim targets 3;

^g^ the average relative risk when the outdoor air meets the WHO air quality guideline.

**Table S10 NO_2_-attribuatable pediatric asthma cases for children of different age and gender.**

|  | **NO_2_-associated pediatric asthma cases,**  **Mean (95%UI), thousand** | | | **Contribution of**  **indoor sources** | | |
| --- | --- | --- | --- | --- | --- | --- |
| **Age groups** | **Boys** | **Girls** | **All** | **Boys** | **Girls** | **All** |
| < 6 years | 241 (135, 327) | 146 (85, 194) | 387 (220, 521) | 27% | 23% | 25% |
| 7-11 years | 78 (44, 105) | 50 (29, 67) | 128 (72, 171) | 28% | 24% | 26% |
| 12-17 years | 67 (34, 90) | 57 (28, 77) | 123 (62, 167) | 31% | 32% | 31% |
| All | 385 (212, 522) | 252 (142, 338) | 637 (354, 859) | 28% | 26% | 27% |

**References**

1. Hu Y, Zhao B. Indoor sources strongly contribute to exposure of Chinese urban residents to PM2.5 and NO2. *J Hazard Mater* 2022; **426**: 127829.

2. Wang M, Luo X, Xu S, et al. Trends in smoking prevalence and implication for chronic diseases in China: serial national cross-sectional surveys from 2003 to 2013. *Lancet Respiratory Medicine* 2019; **7**(1): 35-45.

3. Reitsma MB, Reitsma MB, Kendrick PJ, et al. Spatial, temporal, and demographic patterns in prevalence of smoking tobacco use and attributable disease burden in 204 countries and territories, 1990-2019: a systematic analysis from the Global Burden of Disease Study 2019. *Lancet* 2021; **397**(10292): 2337-60.

4. National Bureau of Statistics of China. Tabulation on the 2010 population census of the People's Republic of China (in Chinese). Beijing: China Statistics Press; 2012. http://www.stats.gov.cn/tjsj/pcsj/rkpc/6rp/left.htm
